# Functional Genomic Screen Identifies *Klebsiella pneumoniae* Factors Implicated in Blocking Nuclear Factor κB (NF-κB) Signaling*

**DOI:** 10.1074/jbc.M114.621292

**Published:** 2015-05-13

**Authors:** Anna Tomás, Leticia Lery, Verónica Regueiro, Camino Pérez-Gutiérrez, Verónica Martínez, David Moranta, Enrique Llobet, Mar González-Nicolau, Jose L. Insua, Juan M. Tomas, Philippe J. Sansonetti, Régis Tournebize, José A. Bengoechea

**Affiliations:** From the a Infection and Immunity Program, Fundación de Investigación Sanitaria de las Islas Baleares (FISIB), 07110 Mallorca, Spain,; the b Instituto de Investigación Sanitaria de Palma (IdisPa), 07120 Mallorca, Spain,; the c Centro de Investigación Biomédica en Red Enfermedades Respiratorias (CIBERES), 28029 Madrid, Spain,; the d Unité de Pathogénie Microbienne Moléculaire, Institut Pasteur, 75724 Paris, France,; e INSERM U786, 75724 Paris, France,; the f Centre for Infection and Immunity, Queen's University Belfast, Belfast BT9 7AE, United Kingdom,; the g Departamento de Microbiología, Facultad de Biología, Universidad de Barcelona, 08028 Barcelona, Spain,; h Chaire de Microbiologie et Maladies Infectieuses, Collège de France, 75231 Paris, France,; i Imagopole, Plateforme d'Imagerie Dynamique, Institut Pasteur, 75724 Paris, France, and; the j Consejo Superior de Investigaciones Científicas (CSIC), 28008 Madrid, Spain

**Keywords:** iron, *Klebsiella pneumonia*, lipopolysaccharide (LPS), NF-κ B (NF-κB), toll-like receptor (TLR), pullulanase

## Abstract

*Klebsiella pneumoniae* is an etiologic agent of community-acquired and nosocomial pneumonia. It has been shown that *K. pneumoniae* infections are characterized by reduced early inflammatory response. Recently our group has shown that *K. pneumoniae* dampens the activation of inflammatory responses by antagonizing the activation of the NF-κB canonical pathway. Our results revealed that *K. pneumoniae* capsule polysaccharide (CPS) was necessary but not sufficient to attenuate inflammation. To identify additional *Klebsiella* factors required to dampen inflammation, we standardized and applied a high-throughput gain-of-function screen to examine a *Klebsiella* transposon mutant library. We identified 114 mutants that triggered the activation of NF-κB. Two gene ontology categories accounted for half of the loci identified in the screening: metabolism and transport genes (32% of the mutants) and envelope-related genes (17%). Characterization of the mutants revealed that the lack of the enterobactin siderophore was linked to a reduced CPS expression, which in turn underlined the NF-κB activation induced by the mutant. The lipopolysaccharide (LPS) O-polysaccharide and the pullulanase (PulA) type 2 secretion system (T2SS) are required for full effectiveness of the immune evasion. Importantly, these factors do not play a redundant role. The fact that LPS O-polysaccharide and T2SS mutant-induced responses were dependent on TLR2-TLR4-MyD88 activation suggested that LPS O-polysaccharide and PulA perturbed Toll-like receptor (TLR)-dependent recognition of *K. pneumoniae*. Finally, we demonstrate that LPS O-polysaccharide and *pulA* mutants are attenuated in the pneumonia mouse model. We propose that LPS O-polysaccharide and PulA T2SS could be new targets for the design of new antimicrobials. Increasing TLR-governed defense responses might provide also selective alternatives for the management of *K. pneumoniae* pneumonia.

## Teaser

**Background:** There is limited knowledge of *Klebsiella pneumoniae* determinants implicated in the blocking of the NF-κB signaling pathway.

**Results:** A high-throughput genetic screen led to the identification of 114 putative *K. pneumoniae* genes that are associated with suppression of NF-κB activation.

**Conclusion:** CPS, LPS, and the T2SS-secreted PulA are needed for immune evasion.

**Significance:** A new therapeutic approach to treat *Klebsiella* infections will be the prevention of immune evasion.

## Introduction

*Klebsiella pneumoniae* is a Gram-negative pathogen causing a wide range of infections from urinary tract infections to pneumonia. *Klebsiella*-triggered pneumonia is particularly devastating among immunocompromised patients with mortality rates between 25 and 60% (1). *K. pneumoniae* is a member of the so-called ESKAPE group of microorganisms to emphasize that they effectively “escape” the effects of antibacterial drugs (2). Despite the clinical relevance of *K. pneumoniae*, the infection biology of this pathogen is poorly understood, and therefore it is both urgent and necessary to better understand its pathophysiology to be able to design new strategies to treat *K. pneumoniae* infections.

The best characterized virulence factor of this pathogen is the capsule polysaccharide (CPS).^3^ Isogenic CPS mutant strains are avirulent and unable to cause pneumonia and urinary tract infections (3–5). Lipid A of lipopolysaccharide (LPS), the outer membrane proteins OmpA and OmpK36, iron-scavenging systems, and several adhesins are other virulence determinants that have been characterized (6–10). Nonetheless, there is still limited knowledge of the exact role of individual virulence factors in *Klebsiella* infections.

A wealth of evidence indicates that the activation of early inflammatory responses is essential to clear *Klebsiella* infections (11–14). Any interference with this response leads to a more severe infection (15), thus in turn, augmenting the immune response with exogenous inflammatory mediators decreases the mortality associated with *K. pneumoniae* infection (16–19). Collectively, this evidence suggests that *Klebsiella* tries to counteract the induction of these host defense responses. Indeed, we (8, 20–22) and others (23) have provided compelling evidence for this notion.

At the molecular level, we have demonstrated that *K. pneumoniae* abrogates the activation of inflammatory responses by targeting NF-κB and MAPK signaling pathways (22, 24). *Klebsiella* antagonizes the activation of NF-κB via the deubiquitinase CYLD and blocks the phosphorylation of MAPKs via the MAPK phosphatase MKP-1 (22). CYLD and MKP-1 are normally regulated to return to homeostasis after inflammation to protect the host from an overwhelming inflammatory response (25, 26). To exert this anti-inflammatory effect, *K. pneumoniae* affects the membrane association of the receptor NOD1 (22). This is dependent on *Klebsiella*-triggered Rho GTPase Rac1 inhibition (22). To identify additional host factors involved in the anti-inflammatory effect, we applied an unbiased high-throughput siRNA gain-of-function screen to interrogate the human kinome (24). Follow-up validation revealed that *K. pneumoniae* exploits an EGF receptor (EGFR)-PI3K-AKT-PAK4-ERK-GSK3β signaling pathway to induce the expression of the deubiquitinase CYLD to attenuate the cytokine-dependent nuclear translocation of NF-κB (24). Our group uncovered a role for CPS in the activation of EGFR and EGFR-dependent signaling (24). However, because CPS does not activate NOD1-dependent responses (22), *K. pneumoniae* may employ additional factors to attenuate NF-κB activation.

This study was designed to identify *K. pneumoniae* determinants implicated in blocking the activation of the NF-κB signaling pathway. To take a systematic approach toward the identification of these bacterial factors, we performed a high-throughput genetic screen to mine a transposon mutant library of *K. pneumonia* strain 52145. This is a reference strain of serotype K2 highly virulent *Klebsiella* strains from which important virulence factors, including the large virulence plasmid harboring the regulator of mucoid phenotype (*rmpA*) and the aerobactin cluster, have been identified (27). We found 114 mutants that, in sharp contrast to the wild-type strain, activated the NF-κB signaling pathway. Further characterization confirmed the critical role of K. *pneumoniae* CPS in blocking NF-κB activation and uncovered the role of the LPS polysaccharide section and the pullulanase type II secretion system (T2SS) in immune evasion.

## Experimental Procedures

### Bacterial Strains, Growth Conditions, and Reagents

Strains and plasmids used in this study are listed in Table 1. *K. pneumoniae* 52145 (hereafter Kp52145) is a clinical isolate (serotype O1:K2) described previously (3, 28). Bacteria were grown in Luria-Bertani (LB) medium at 37 °C unless indicated otherwise. When appropriate, antibiotics were added to the growth medium at the following concentrations: ampicillin, 100 μg/ml; rifampicin, 50 μg/ml; kanamycin, 50 μg/m; cloramphenicol (Cm), 25 μg/ml; and trimethoprim (Tp), 100 μg/ml. FeSO_4_, and FeCl_3_ were used at a concentration of 10 μm, and 2-2′-dipyridyl (Sigma) was used at a concentration of 200 μm. The sequences of the primers used in this study are available from the authors upon request.

**TABLE 1:** **Bacterial strains and plasmids used in this study**

Bacterial strains and plasmids	Genotype or comments	Source or reference
**Strains**		
*Escherichia coli*		
CC118-λpir	Δ(ara-leu)7967 araD139 ΔlacX74 *galE galK* ΔphoA20 thi-1 *rps*E *rpoB argE*(Am) *recA1*	Laboratory collection
DH5a-λpir	DlacU169 (F80lacZDM15) *recA1 endA1 hsdR17* thi-1 *gyrA96 relA1* λpir phage lysogen	Laboratory collection
SY327-λpir	(lac pro) *argE* (Am) *rif nalA recA56* (λpir)	Laboratory collection
*Klebsiella pneumoniae*		
Kp52145	Clinical isolate; serotype O1:K2; Rif^R^	(3, 28)
52145-Δ*wca*_K2_	Kp52145, Δwca_K2_, capsule mutant; Rif^R^	(32)
52OmpA2	Kp52145, Δ*ompA*::Cm, *ompA* gene is inactivated; Rif^R^, Cm^R^	(37)
52Δ*wcaK2*OmpA	52145-Δwca_K2_, Δ*ompA*::Cm, capsule mutant in which *ompA* is inactivated; Rif^R^, Cm^R^	(37)
52ΔwaaL	Kp52145, Δ*waaL*, *waaL* is inactivated; nonpolar mutant; Rif^R^	(68)
52ΔwabM	Kp52145, Δ*wabM*, *wabM* is inactivated; nonpolar mutant; Rif^R^	(50)
52ΔwabH	Kp52145, Δ*wabH*, *wabH* is inactivated; nonpolar mutant; Rif^R^	(50)
52ΔwabK	Kp52145, Δ*wabK*, *wabK* is inactivated; non polar mutant; Rif^R^	(50)
52145-Δwca_K2_ΔwaaL	52145-Δwca_K2_; Δ*waaL*, *waaL* is inactivated; non polar mutant; Rif^R^	(6)
52145-Δwca_K2_ΔwabM	52145-Δwca_K2_; Δ*wabM*, *wabM* is inactivated; non polar mutant; Rif^R^	(6)
52145-Δwca_K2_ΔwabK	52145-Δwca_K2_; Δ*wabK*, *wabK* is inactivated; non polar mutant; Rif^R^	(6)
52145-Δwca_K2_ΔwabH	52145-Δwca_K2_; Δ*wabH*, *wabH* is inactivated; non polar mutant; Rif^R^	(6)
52waaL::tn*5*	Kp52145; Δ*waaL*::tn*5*, *waaL* is inactivated; Rif^R^, K_m_^R^	This study
52entF::tn*5*	Kp52145; *entF*::tn*5*, *entF* is inactivated; Rif^R^, K_m_^R^	This study
52145-Δwca_K2_entF	52145-Δwca_K2,_ *entF*:: pKNOCK-Cm_entF, capsule mutant in which *entF* is inactivated; Rif^R^, Cm^R^	This study
52pulC::tn*5*	Kp52145; ΔpulC::tn*5*, *pulC* is inactivated; Rif^R^, K_m_^R^	This study
52irp1	Kp52145, *irp1*:: pKNOCK-Cm_IRP1, *irp1* is inactivated; Rif^R^, Cm^R^	This study
52iutA	Kp52145, *iutA*:: pKNOCK-Cm_iutA, *iutA* is inactivated; Rif^R^, Cm^R^	This study
52glf	Kp52145, *glf*:: pSFglf, *glf* is inactivated; Rif^R^, K_m_^R^	This study
52ΔpulA	Kp52145, *pulA*:: pKNOCK-Cm_pulA, *pulA* is inactivated; Rif^R^, Cm^R^	This study
52pulACom	52ΔpulA harboring mini-Tn7TKmKpnpulA; PulA levels restored; Rif^R^, Cm^R^, K_m_^R^	This study
52145-Δwca_K2_pulA	52145-Δwca_K2,_ *pulA*:: pKNOCK-Cm_pulA, capsule mutant in which *pulA* is inactivated; Rif^R^, Cm^R^	This study
52145-Δwca_K2_pulACom	52145-Δwca_K2_pulA mutant harboring mini-Tn7TKmKpnpulA; PulA levels restored; Rif^R^, Cm^R^, K_m_^R^	This study
52ΔwaaLpulA	52ΔwaaL, *pulA*:: pKNOCK-Cm_pulA, *waaL* mutant in which *pulA* is inactivated; RifR, CmR	This study
52145-Δwca_K2_ΔwaaL pulA	52145-Δwca_K2_ΔwaaL, *pulA*:: pKNOCK-Cm_pulA, capsule and *waaL* mutant in which *pulA* is inactivated; RifR, CmR	This study

**Plasmids**		
pRL27	Tn*5* transposon delivery vector, K_m_^R^	(30)
pGEM-T Easy	Cloning plasmid, Amp^R^	Promega
pKNOCK-Cm	Suicide vector, R6K replication origin, Cm^R^	(35)
pKNOCK-Cm_entF	pKNOCK-Cm containing an internal fragment of *entF* gene for mutant construction, Cm^R^	This study
pKNOCK-Cm_IRP1	pKNOCK-Cm containing an internal fragment of *irp1* gene for mutant construction, Cm^R^	This study
pKNOCK-Cm_iutA	pKNOCK-Cm containing an internal fragment of *iutA* gene for mutant construction, Cm^R^	This study
pKNOCK-Cm_pulA	pKNOCK-Cm containing an internal fragment of *pulA* gene for mutant construction, Cm^R^	This study
pSF100	Suicide vector, R6K replication origin, K_m_^R^	(36)
pSFglf	pSF100 containing an internal fragment of *glf* gene for mutant construction, K_m_^R^	This study
pUC18R6KT-mini-Tn7TKm	pUC18R6KT-mini-Tn7TKm containing a K_m_ cassette, Amp^R^, K_m_^R^	(37)
pTSNSK-Tp	pTSNSK-Tp containing a transposase for Tn7 insertion, K_m_^R^, Tp^R^	(38)
pUC18R6KT-mini-Tn7TKm_pulACom	pUC18R6KT-mini-Tn7TKm containing *pulA* gene for complementation, Amp^R^, K_m_^R^	This study
p34S-Tp	Source of Tp cassette; Amp^R^, Tp^R^	(40)
pPROBE′-gfp[LVA]	Plasmid containing *gfp* without promoter as reporter gene, K_m_^R^	(41)
pPROBE′-gfp[LVA]Tp	Trimethoprim resistance cassette cloned into SphI site of pPROBE′-gfp[LVA], Tp^R^	This study
pPROBE′-gfp[LVA]Tp_entC	pPROBE′-gfp[LVA]Tp containing *entC* promoter region, Tp^R^	This study
pPROBE′-gfp[LVA]Tp_ybtA	pPROBE′-gfp[LVA]Tp containing *ybtA* promoter region, Tp^R^	This study
pPROBE′-gfp[LVA]Tp_psn	pPROBE′-gfp[LVA]Tp containing *psn* promoter region, Tp^R^	This study
pPROBE′-gfp[LVA]Tp_iucA	pPROBE′-gfp[LVA]Tp containing *iucA* promoter region, Tp^R^	This study
pGPLKpnCps	pGPL01 containing *cps* promoter region; Amp^R^	(37)

### Cell Culture and Infection

Monolayers of A549 (ATCC CCL185) were grown as described previously (22). For infections, A549 cells were seeded to 90% confluence (3 × 10^5^ cells/well) in 24-well tissue culture plates. Cells were serum-starved for 16 h before infection. Bacteria were prepared as described (22), and infections were performed using a multiplicity of infection of 100 bacteria/cell unless indicated otherwise. For incubation times longer than 120 min, bacteria were killed by the addition of gentamicin (100 μg/ml), which was not removed until the end of the experiment. Cell viability, assessed by trypan blue dye exclusion, was >95% even after 4 h of infection.

### Mutant Library Construction

Kp52145 was made electrocompetent following the method described by Sharma and Schimke (29). Transposon mutagenesis was performed by electroporating ∼1 μg of the pRL27 mini Tn5 transposon (30) to 50 μl of Kp52145 electrocompetent cells using a Gene Pulser Xcell (Bio-Rad) followed by a 60-min recovery in SOC (super optimal broth) medium at 37 °C. The transposon carries a kanamycin resistance cassette and an R6K origin of replication (30). Transposants were plated on LB-rifampicin-kanamycin plates, and the transposon mutant library was generated from six independent electroporation rounds. A total of 5320 mutants were arrayed in a 96-well plate format and stored at −80 °C in 20% (v/v) glycerol (master plate). One well without bacteria was kept as a negative control in all plates (typically well H12). Random transposon insertion was checked in ∼200 clones by direct genomic sequencing. Genomic DNA was extracted using the Realpure spin kit (Real). Sequencing reaction was performed in an iCycler (Bio-Rad) containing 2 μl of BigDye Master Mix, 2 μl of 5× buffer, 1 μl of betaine (5 m), 1 μm primer (tpnRL17), and 2–5 μg of genomic DNA. The thermal profile consisted of 5 min of a denaturalization step at 95 °C followed by 100 cycles of 95 °C for 30 s, 50 °C for 20 s, and 60 °C for 4 min. Sequencing reactions were purified following the manufacturer's instructions, resuspended in 10 μl of Hi-Di formamide, and run in an ABI 3730 genome sequencer (Applied Biosystems).

### Construction of a NF-κB Reporter Cell Line

A549 cells were seeded into 24-well plates at a density of 4 × 10^4^ cells/well and transfected 24 h later with 5 μg of pNifty2-SEAP (InvivoGen) using Lipofectamine 2000 (Invitrogen) lipofection reagent according to the manufacturer's instructions. In the pNifty2-SEAP vector, the proximal promoter of the endothelial cell-leukocyte adhesion molecule (ELAM-1; E-selectin), containing three NF-κB sites and lacking an AP1/CREB (cAMP-response element-binding protein) site found in the full-length promoter, controls the reporter gene, SEAP (secreted form of the human embryonic alkaline phosphatase). Twenty-four hours after transfection, cells were selected with 500 μg/ml Zeocin for 2 weeks (InvivoGen). Cells were singularized by serial dilutions in 96-well plates, and monoclonal cell lines were propagated for 2 weeks. Stably transfected A549-SEAP cells were grown in complete RPMI medium supplemented with 100 μg/ml Zeocin. A2 was the clone selected for screening purposes.

### High-throughput Screening

#### 

##### Cell Seeding and Bacterial Culture

A549-SEAP A2 cells from the same passage number were seeded into 96-well plates at 1–2 × 10^4^ cells/well in 100 μl of RPMI 1640. Transposon mutants and controls were grown in 96-well plates. 5 μl from the bacteria master plate were used to inoculate 150 μl of LB supplemented with the appropriate antibiotics. Plates were incubated overnight at 37 °C without shaking. Bacterial growth was determined by measuring absorbance at 600 nm (*A*_600_) using a microplate reader (Biotek PowerWave HT).

##### Infection

A549-SEAP A2 cells were washed twice with 200 μl of PBS, and then 150 μl of RPMI 1640 supplemented with 10% fetal calf serum (FCS) was added. Five μl of the bacterial overnight culture was used to infect the cells (multiplicity of infection, ∼100:1). Only the inner 60 wells of the plate were used. Two replicates of each control (negative/positive (IL-1β, 10 ng/ml) cells infected with Kp52145, 52OmpA, 52145Δwca_K2_, and 52145Δwca_K2_-OmpA) and 48 mutants were run in each plate. After 3 h, wells were washed twice with 200 μl of PBS, and then 100 μl of RPMI 1640 containing 100 μg/ml of gentamicin was added to each well. Plates were incubated overnight in a humidified CO_2_ incubator.

##### Colorimetric Assay

30 μl of the supernatant was mixed with 200 μl of QUANTI-Blue reagent (InvivoGen), and the mixture was incubated at 37 °C for 24 h. The levels of SEAP were measured at an absorbance at 625 nm (*A*_625_).

##### Statistical Analysis

The *Z* score was calculated for each of the 48 wells of a plate as described previously (31). For a given well, the *Z* score was calculated by subtracting the mean value of the wells on that plate from the value of the well and dividing by the standard deviation value for all the plate wells. A *Z* score ≥ 2 was considered significant. As means and standard deviations are greatly influenced by statistical outliers (31), which in the context of a screening are putative hits, we also selected mutants that induced SEAP levels higher than *A*_625_ > 0.5, which represents a 2-fold *A*_625_ value induced by the wild-type strain.

##### Hit Validation

Four independent validation rounds were run for each selected hit. A hit was considered validated if the SEAP levels were *A*_625_ > 0.5 in at least two of four validation runs.

##### Transposon Insertion Site Identification

Genomic DNA was purified using the Realpure spin kit (Real). Direct genomic sequencing was carried out as described above (see “Mutant Library Construction”).

##### Growth Deficiency Determination

To identify those hits with growth deficiencies, transposon mutants were plated on LB plates or M9 plates supplemented with either 0.2% glucose or 10 mm citrate and incubated at 37 °C for 48 h.

### CPS Purification and Quantification

Cell-associated CPS from *K. pneumoniae* strains were purified using the hot phenol-water method exactly as described previously (32). CPS was quantified by determining the concentration of uronic acid in the samples using a modified carbazole assay (33).

### LPS Extraction and Analysis by SDS-PAGE

The LPS from *K. pneumoniae* strains was extracted by a modified phenol-water purification method (34). The LPS was run on a 12% SDS-PAGE and visualized using a Pro-Q Emerald 300 lipopolysaccharide gel stain Kit (Invitrogen).

### K. pneumoniae Mutant Construction

The *entF*, *irp1*, *iutA*, and *pulA* mutants were constructed by insertion mutagenesis using the pKNOCK-Cm suicide vector (35). An internal fragment of each gene was amplified using genomic DNA from wild-type *K. pneumoniae* 52145, *Vent* polymerase (New England Biolabs), and the corresponding primer pairs. PCR products were phosphorylated and cloned into the SmaI-digested and dephosphorylated pKNOCK-Cm vector to obtain pKNOCK-Cm_entF, pKNOCK-Cm_irp1, pKNOCK-Cm_iutA, and pKNOCK-Cm_pulA, respectively. The *glf* mutant was also constructed by insertion mutagenesis using the *pir* replication-dependent plasmid pSF100 (36). Plasmids were introduced into *Klebsiella* by conjugation and PCR, or Southern blot was used to identify strains in which plasmids were inserted into the genome by homologous recombination.

### Complementation of pulA Mutant

To complement the *pulA* mutant, a DNA fragment of 4,008 bp containing the putative promoter region and coding region of *pulA* was PCR-amplified (primer pair pulACom.F-pulACom.R) using *Vent* polymerase, gel-purified, and cloned into SmaI-digested pUC18R6KT-mini-Tn7TKm (37) to obtain pUC18R6KT-mini-Tn7TKm_pulACom. The pTSNSK-Tp plasmid, which contains the transposase *tnsABCD*, necessary for Tn7 transposon mobilization (38), was electroporated into the *pulA* mutant. Then, the pUC18R6KT-mini-Tn7TKm_pulACom plasmid was mobilized into this strain by triparental conjugation using the helper strain *Escherichia coli* HB101/pRK2013. Colonies were screened for resistance to kanamycin and sensitivity to ampicillin. Because the ampicillin cassette is located outside of the Tn7 region on the vector, sensitivity to ampicillin denotes the integration of the Tn7 derivative at the *attTn7* site instead of incorporation of the vector into the chromosome. Confirmation of integration of the Tn7 transposon at the established *attTn7* site located downstream of the *glmS* gene was verified by a multiplex PCR using primers glmS.UP, glmS.DW, Tn7.L, and Tn7.R. PCR reactions were performed in a final volume of 25 μl containing 50 ng of DNA, 1.5 mm MgCl_2_, 0.2 mm dNTPs, each primer at 0.2 μm, and 1 unit of Go*Taq* polymerase (Promega). The thermal profile was as follows: an initial denaturation step at 94 °C for 5 min followed by 5 cycles of 94 °C for 45 s, a touchdown from 62 to 58 °C for 45 s and 72 °C for 45 s, and then 25 cycles of 94 °C for 45 s, 58 °C for 45 s, and 72 °C for 45 s, and a final extension step at 72 °C for 5 min. Correct insertion of the Tn7 transposon yielded two amplicons of 462 and 216 bp amplified with primer pairs glmS.UP-Tn7.L and glmS.Dw-Tn7.R, respectively. Only one amplicon of 455 bp, corresponding to an internal fragment of the *glmS* gene, was amplified when used as the template DNA from wild-type *Klebsiella*. pTSNSK-Tp from the recipient strain was cured by growing bacteria at 37 °C due to the plasmid-thermosensitive origin of replicationpSC101. Plasmid removal was confirmed by susceptibility to trimethoprim.

PulA levels on the outer membrane fraction, purified as described previously (39), were detected by immunoblotting using rabbit antibody anti-PulA (1:1000), kindly donated by A. Pugsley. Proteins were resolved using 12% SDS-PAGE and electroblotted to nitrocellulose membrane. Membranes were blocked with 4% skim milk in TBST (137 mm NaCl, 2.7 mm KCl, 19 mm Tris base (pH 7.4)).

### Construction of gfp Reporter Fusions

A trimethoprim resistance cassette was obtained as a SmaI fragment from plasmid p34S-Tp (40). The cassette wascloned into SphI-digested and blunt-ended plasmid pPROBE′-gfp[LVA] to obtain pPROBE′-gfp[LVA]Tp. This vector contains a promoterless *gfp* gene (41). DNA fragments containing the promoter regions of *entC*, *ybtA*, *psn*, and *iucA* were amplified by PCR using *Vent* polymerase, gel-purified, and cloned into SmaI-digested pPROBE′-gfp[LVA]Tp plasmid. Plasmids were introduced in *E. coli* DH5α-λpir and then mobilized into *Klebsiella* by triparental conjugation using the helper strain *E. coli* HB101/pRK2013.

### Luciferase Activity and GFP Fluorescence Measurements

Overnight cultures of the reporter strains were diluted 1/10 in fresh LB medium and incubated for 3 h at 37 °C on an orbital incubator shaker (180 rpm). The cultures were harvested(4000 × *g* for 20 min) and resuspended to an *A*_600_ of 1.0 in PBS. Luciferase activity and GFP fluorescence were determined as described previously and expressed as relative light units (RLU) or relative fluorescence units (RFU) (42). All measurements were performed in triplicate on at least three independent occasions.

### Adhesion and Internalization Assays

Bacteria adhesion and internalization to A549 cells were determined as our group described previously (8). The results are expressed as cfu/well. Experiments were carried out in duplicate on at least three independent occasions.

### IL-8 Stimulation Assay

A549 cells, seeded in 24-well plates, were infected for 2 h and then washed twice with PBS, and fresh medium plus gentamicin (100 μg/ml) was added. Supernatants were recovered after 12 h, cell debris was removed by centrifugation, and samples were frozen at −80 °C. IL-8 levels in the supernatants were determined using a commercial ELISA (eBioscience) with a sensitivity of <2 pg/ml. Experiments were run in duplicate and repeated at least three independent times.

### Inmunoblotting

Eukaryotic proteins were resolved by standard 10% SDS-PAGE and electroblotted onto nitrocellulose membranes. Membranes were blocked with 4% skim milk in TBST, and protein bands were detected with specific antibodies using chemiluminescence reagents and a GeneGnome chemiluminescence imager (Syngene). Immunostainings for IκBα were performed using polyclonal rabbit anti- IκBα (1:1,000, Cell Signaling) antibodies. Immunostainings to assess phosphorylation of p38, p44/42, and JNK MAPKs were performed using polyclonal rabbit, anti-phospho-p38, anti-phospho-p44/42, and anti-phospho-JNK antibodies, respectively (all used at 1:1000; Cell Signaling). Blots were reprobed with the polyclonal antibody anti-human tubulin (1:3000, Sigma) to control that equal amounts of proteins were loaded in each lane.

### Small Interfering RNA (siRNA)

Transfection of siRNAs was performed at the time of cell seeding in a 96-well plate format (2 × 10^4^ cells/well). Lipofectamine^TM^ 2000 transfection reagent (Invitrogen) was used following the manufacturer's instructions. Transfection experiments were carried out in Opti-MEM reduced serum medium (Invitrogen). siRNAs were used at a concentration of 20 nm, and experiments were carried out 48 h after transfection. The knockdown efficiency of the siRNAs targeting NOD1, TLR2, TLR4, and EGFR was verified previously (22, 24).

### Murine Infection Model

Mice were treated in accordance with the Directive of the European Parliament and the Council on the Protection of Animals Used for Scientific Purposes (Directive 2010/63/EU) and in agreement with the Bioethical Committee of the University of the Balearic Islands. This study was approved by the Bioethical Committee of the University of the Balearic Islands (authorization number 1748).

Five-to-seven-week-old female CD-1 mice (Harlan) were infected as described previously by our group (8). Non-infected mice were inoculated with 20 μl of PBS. At 24 h post-infection, mice were euthanized by cervical dislocation. Tissues were rapidly dissected for bacterial load determination, and half of the lungs were immediately frozen in liquid nitrogen and stored at −80 °C until purification of RNA was carried out.

### RNA Purification and Real Time Quantitative PCR (RT-qPCR)

RNA from lungs was purified as described by us (8). cDNA was obtained by retrotranscription of 1 μg of total RNA using the M-MLV reverse transcriptase (Sigma). The reaction included one step to eliminate traces of genomic DNA. RT-qPCR analyses were performed using an iCycler real-time PCR instrument (Bio-Rad). Reactions were performed in 25 μl containing 12.5 μl of KAPA SYBR® FAST qPCR kit master mix (KapaBiosystems), each primer at 0.2 μm, and 50 ng of cDNA. Relative quantities of mRNAs were obtained using the comparative threshold cycle (ΔΔC_T_) method by normalizing to *hprt1*.

Bacteria were grown at 37 °C in 5 ml of LB medium on an orbital incubator shaker (180 rpm) until an *A*_600_ of 0.3 was reached. RNA was purified following an established protocol (43). cDNA was obtained by retrotranscription of 2 μg of total RNA using commercial M-MLV reverse transcriptase (Sigma) and random primers mixture (SABiosciences, a Qiagen company). 50 ng of cDNA was used as a template in a 25-μl reaction. RT-PCR analyses were performed with a Smart Cycler real-time PCR instrument (Cepheid, Sunnyvale, CA) using a KapaSYBR Fast qPCR kit as recommended by the manufacturer (Cultek). SYBR Green dye fluorescence was measured at 521 nm. cDNAs were obtained from two independent extractions of mRNA, and each one was amplified by RT-qPCR on two independent occasions. Relative quantities of *wabM* mRNAs were obtained using the comparative threshold cycle (ΔΔC_T_) method by normalizing to the *rpoD* gene.

### Statistical Analysis

Results were analyzed by analysis of variance (ANOVA) or with a one-tailed *t* test followed by a Bonferroni contrast correction for multiple testing using GraphPad Prism software (GraphPad Software Inc.). Results are given as the means ± S.D. A *p* value of <0.05 was considered statistically significant.

## Results

### 

#### 

##### High-throughput Screening to Identify K. pneumoniae Loci Involved in Attenuating NF-κB Activation

**FIGURE 1.**
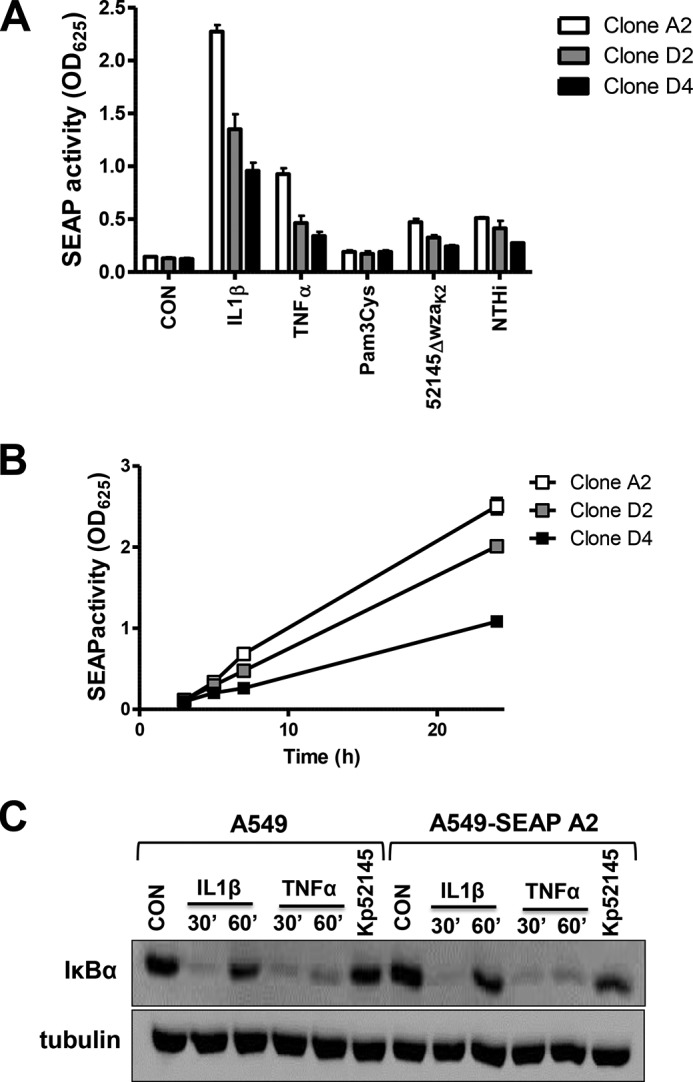
**Construction of a NF-κB reporter cell line.** *A*, SEAP levels released by three clones of A549 cells stably transfected with NF-κB reporter pNifty2-SEAP vector. Cells were left untreated (control (*CON*), *white bars*), treated with IL-1β (10 ng/ml), TNFα (10 ng/ml), or TLR2 agonist Pam3Cys (100 ng/ml), or infected with *Klebsiella* capsule mutant (52145-Δ*wza*_K2_) or nontypable *Haemophilus influenzae* 398 (*NTHi*). *Scale bars* represent mean ± S.E. (*n* = 3). *B*, time course of SEAP levels released by the selected clones treated with IL-1β (10 ng/ml) (*n* = 3). *C*, immunoblot analysis of IκBα and tubulin levels in lysates of A549 and clone A549-SEAP A2 cells were left untreated (control), treated with IL-1β (10 ng/ml) or TNFα (50 ng/ml) for the indicated time, or infected for 3 h with Kp52145. Data are representative of three independent experiments.

We developed an assay suitable for identifying the *K. pneumoniae* loci required to block activation of the NF-κB pathway. A new indicator cell line was engineered by transfection of the human epithelial cell line A549 with the reporter vector pNIFTy2-SEAP (InvivoGen). Three clones were selected, A549-SEAP A2, D2, and D4, and control experiments were carried out to determine whether different stimuli known to activate NF-κB induce the secretion of SEAP (Fig. 1*A*). Clone A2 secreted higher amounts of SEAP than the other two clones after cells were challenged with different stimuli (Fig. 1*A*). Time course experiments showed that clone A2 secreted higher levels of SEAP over time than the other two clones upon challenge with IL-1β (10 ng/ml) (Fig. 1*B*). Western blot analysis of IκBα levels showed no differences between A549 cells and A549-SEAP A2 after stimulation with different agonists (Fig. 1*C*). Clone A2 was selected for screening purposes.

**FIGURE 2.**
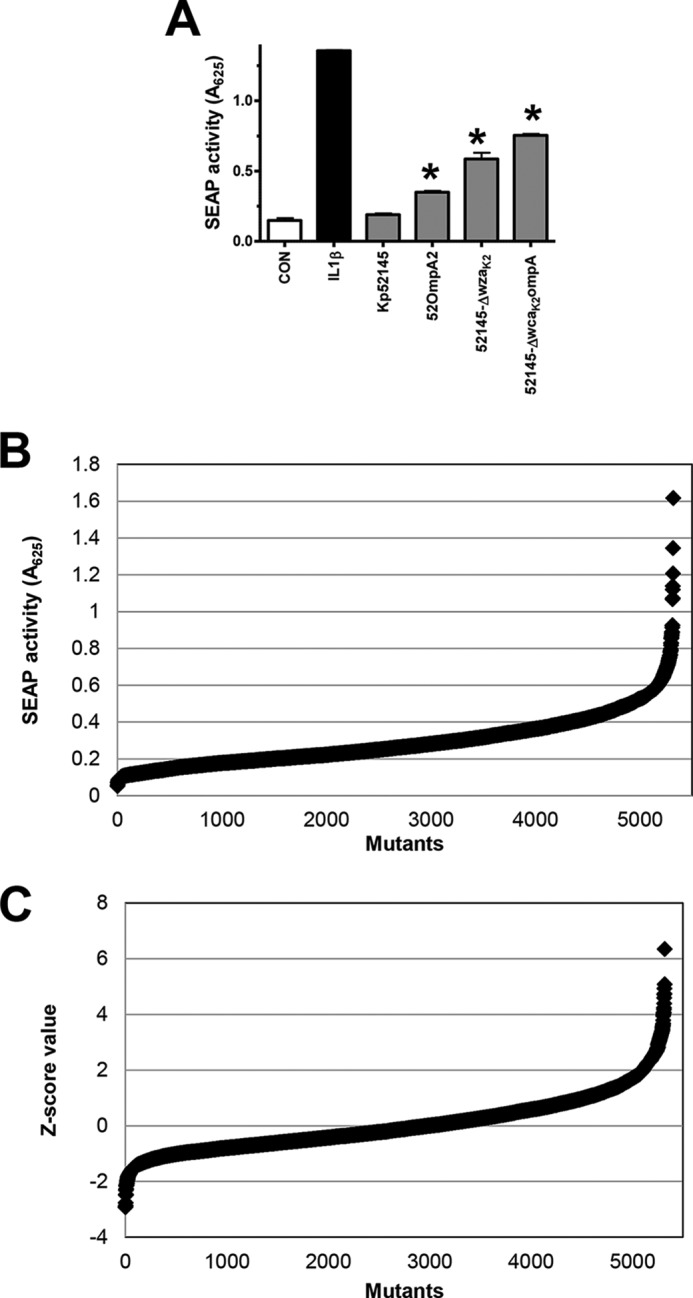
**High-throughput screening for the identification of *K. pneumoniae* factors implicated in attenuation of NF-κB activation.** *A*, SEAP levels released by A549-SEAP A2 cells left untreated (control (*CON*), *white bars*), treated with IL-1β (10 ng/ml) (*black bars*), or infected with Kp52145, 52OmpA2, 52145-Δ*wza*_K2_, or 52145-Δ*wca*_K2_ompA (*gray bars*). *Scale bars* represent mean ± S.E. (*n* = 3) *, *p* < 0.05 (results are significantly different from the results for Kp52145; one-tailed *t* test). *B*, distribution of SEAP levels released by A549-SEAP A2 cells infected with the mutants. *C*, distribution of *Z* score data of SEAP levels.

SEAP levels induced by Kp52145 were not significantly different than those observed in non-infected cells (Fig. 2*A*). In contrast, the *cps*, *ompA* mutants, and the *cps-ompA* double mutant did trigger the secretion of SEAP. We have already reported that these strains elicit the activation of NF-κB (8). The differences observed between strains are consistent with data from our laboratory showing the relative importance of CPS and OmpA to attenuate NF-κB activation (8).

The assay was then transferred from a 24-well format to a 96-well format by optimizing culture conditions, cell number/well, multiplicity of infection, time of contact, and time of detection of secreted SEAP. To determine the quality of the assay, the *Z*′ factor (31) was calculated taking as a negative control SEAP levels secreted by cells infected with the wild-type strain and as a positive control SEAP levels secreted by cells infected with the *ompA* mutant or cells stimulated with IL-1β. In our case, the resulting *Z*′ factors were 0.73 and 0.86 for the respective comparisons. The *Z*′ factor is a screening window coefficient indicating the capability of a hit identification for a given assay at the defined screening conditions. *Z*′ factors higher than 0.5 indicate that an assay is robust enough for a high-throughput screening (31).

To identify *Klebsiella* loci involved in attenuating NF-κB activation, we examined a bank of 5320 transposon mutants. Each mutant was tested individually. For candidate selection the standard *Z* score normalization procedure was applied and mutants with a *Z* score ≥ 2 were selected. We also selected those mutants that induce SEAP levels higher than *A*_625_ > 0.5, which represents a 2-fold increase in the SEAP levels induced by the wild type. The distribution of *A*_625_ values and the *Z* scores of the mutant library are shown in Fig. 2, *B* and *C*. Following the first round of screening, 522 mutants (10% of the transposon library) were considered candidates for validation.

To validate the selected candidates, each one was tested individually in four independent screening rounds. Candidates were considered validated if they induced SEAP levels higher than *A*_625_ > 0.5 in at least two of four independent experiments upon infection of the A549-SEAP A2 cells. Using this validation criterion, 114 Kp52145 mutants were considered validated (Table 2). Control experiments showed that all mutants grew in LB agar plates, LB broth, and RPMI 1640 complete medium, whereas five and three mutants displayed growth deficiencies when cultivated in M9 agar plates supplemented with glucose and citrate, respectively (Table 2).

**TABLE 2:** ***K. pneumoniae* transposon mutants validated in the screening**

Function	Mutant code	Gene	Kp52145 locus tag	MGH78578 locus tag	Growth*^a^*
**Metabolism**					
Oxydoreductases	P017C4	Pyrroline-5-carboxylate reductase	KpST66_1303	KPN_00329	N/N
	P019E9	Putative oxidoreductase	KpST66_1547	KPN_00505	N/Y
	P033F6	Cytochrome bd-I oxidase subunit I	KpST66_2291	KPN_01153	Y/Y
	P037H10	Putative alcohol dehydrogenase *groES*	KpST66_2527	KPN_01466	Y/Y
	P037H6	γ-Aminobutyraldehyde dehydrogenase	KpST66_3019	KPN_01924	Y/Y
	P035C5	Uncharacterized protein conserved	KpST66_3673	KPN_02456	Y/Y
	P018C10	NADH-quinone oxidoreductase subunit F	KpST66_3892	KPN_02674	Y/Y
	P006E12	Putative uncharacterized protein	KpST66_0795	KPN_03309	Y/Y
	P026H10	*cueO*	KpST66_4209	KPN_00131	Y/Y
Carbohydrates	P036D12	NADPH-dependent preQ0 reductase	KpST66_3564	Unknown function	Y/Y
	P043B5	*N*-Acetylmuramic acid 6-phosphate etherase	KpST66_2662	KPN_01553	Y/Y
	P037E12	Deoxyribose-phosphate aldolase	KpST66_2812	KPN_01702	Y/Y
	P029H4	Putative Carboxymuconolactone decarboxylase family protein	KpST66_1120	KPN_03003	Y/Y
	P009G9	Propanediol dehydratase medium subunit *pduD*	KpST66_0902	KPN_03206	Y/Y
	P025D5	Altronate hydrolase	KpST66_0586	KPN_03519	Y/Y
	P006H10	L(+)-tartrate dehydratase subunit β (l-TTD β)	KpST66_0481	KPN_03639	Y/Y
	P040B5	C-terminal of putative uncharacterized protein *yhjS*	KpST66_0231	KPN_03886	Y/Y
Lipids	P025C1	Putative acetyltransferase	KpST66_0118	KPN_03989	Y/Y
	P026E12	Putative acetyltransferase	KpST66_0118	KPN_03989	Y/Y
	P035E6	acyl carrier protein	KpST66_3624	A79E_1666	Y/Y
	P035D1	Glycerol dehydratase, small subunit	KpST66_0621	KPN_03488	Y/Y
	P038H3	Putative uncharacterized protein	KpST66_4890	Unknown function	Y/Y
	P007C12	Triacylglycerol lipase	pKpST66–1_0006	Unknown function	Y/Y
	P038C2	C-terminal region of triacylglycerol lipase	pKpST66–1_0006	Unknown function	Y/Y
Amino acids	P019F6	Dihydroxy-acid dehydratase	KpST66_4589	KPN_04270	N/N
	P037B8	Ketol-acid reductoisomerase	KpST66_4586	KPN_04273	Y/N
Others	P044A12	Uncharacterized protein *ynjA*	KpST66_2352	KPN_01217	Y/Y
	P040B2	Enterobactin synthase component F, *entF*	KpST66_1661	KPN_00605	Y/Y
	P005E9	Cyclic diguanylate phosphodiesterase	KpST66_4039	KPN_02828	Y/Y
	P037G9	Putative amidohydrolase	KpST66_1569	KPN_00523	Y/Y

**Envelope**					
Protein secretion	P044G9	Putative member of ShlA/HecA/FhaA exoprotein family	KpST66_1736	KPN_00676	Y/Y
	P035G10	C-terminal of putative uncharacterized protein ECs0126	KpST66_4210	KPN_00130	Y/Y
	P038F11	Pullulanase secretion envelope *pulC*	KpST66_4179	KPN_00161	Y/Y
	P044E5	Putative hemolysin activator protein	KpST66_1737	KPN_00677	Y/Y
	P020H1	Acriflavine resistance protein A, *acrA*	KpST66_0069	KPN_04039	Y/Y
	P018H5	Auxiliary transport protein, membrane fusion protein (MFP) family	KpST66_3658	Unknown function	Y/Y
	P029G11	Putative plasmid transfer protein	pKpST66–2_0082	Unknown function	Y/Y
Peptidoglycan	P021E7	Penicillin-binding protein 1B	KpST66_4173	KPN_00164	Y/Y
	P035C6	*ampG* muropeptide MFS transporter	KpST66_1369	KPN_00395	Y/Y
	P017C10	Putative β-lactamase-like	KpST66_2943	KPN_01845	Y/Y
Adhesion	P043A12	TPR repeat lipoprotein	KpST66_0546	KPN_03571	Y/Y
	P018G12	Putative fimbrial chaperone protein	KpST66_4961	KPN_04471	Y/Y
	P043D6	*traH*	pKpST66–1_0057	KPN_pKPN4p07153	Y/Y
LPS	P020C10	O-antigen ligase, *waaL*	KpST66_0145	KPN_03966	Y/Y
	P025F5	O-antigen ligase, *waaL*	KpST66_0144	KPN_03966	Y/Y
Others	P050B3	putative OmpA domain protein	KpST66_0945	KPN_03169	Y/Y
	P018C9	Putative outer membrane protein	pKpST66–1_0065	AY703481	Y/Y
	P018F7	Putative outer membrane protein	pKpST66–1_0065	AY703481	Y/Y
	P045A8	Phage tail fiber protein	KpST66_2280	A79E_3050	Y/Y
	P021E9	Putative fusaric acid resistance domain protein	KpST66_3659	KPN2242_15265	Y/Y

**Transport**	P025C6	Putative extracellular solute-binding protein, family 3	KpST66_1626	KPN_00572	Y/Y
	P025D4	Putative extracellular solute-binding protein, family 3	KpST66_1626	KPN_00572	Y/Y
	P007C1	Putative ABC transporter	KpST66_2950	KPN_01850	Y/Y
	P007H1	ABC-type dipeptide/oligopeptide/nickel transport systems, permease component	KpST66_3752	KPN_02536	Y/Y
	P051H4	Putative high-affinity nickel-transporter	KpST66_1081	KPN_03047	Y/Y
	P038C9	d-Galactonate transporter	KpST66_0012	KPN_04094	Y/Y
	P018G8	Spermidine/putrescine import ATP-binding protein *potA*	KpST66_4976	KPN_04456	Y/Y
	P008H2	Putative ascorbate-specific PTS system enzyme IIC	KpST66_4846	KPN_04586	Y/Y
	P034F5	Putative periplasmic-binding protein	KpST66_1095	KPN_03033	Y/Y

**Transcription**	P034C6	Threonyl-tRNA synthetase	KpST66_3559	Unknown function	Y/Y
	P006G10	Transcriptional activator *cadC*	KpST66_1540	KPN_00498	Y/Y
	P019F8	HTH-type transcriptional regulator YcgE	KpST66_1826	KPN_00783	Y/Y
	P009C4	Putative transcriptional regulatory protein TyrR	KpST66_2445	KPN_01305	Y/Y
	P012G4	Putative Mgl repressor	KpST66_4978	KPN_04454	Y/Y
	P041F4	Putative protein YtfJ	KpST66_4824	KPN_04609	Y/Y
	P019G5	Putative TetR family transcriptional regulator	KpST66_3249	A79E_2099	Y/Y
	P038F5	Formylmethionylaminoacyl-tRNA deformylase	pKpST66–2_0076	LV116 (pLVPK)	Y/Y

**Stress**	P049G10	Chaperone *surA*	KpST66_4290	KPN_00050	N/Y
	P046C4	Putative 2-component transcriptional regulator	KpST66_3517	KPN_02412	Y/Y
	P046H11	Protease III	KpST66_0878	KPN_03230	Y/Y
	P025H2	Putative uncharacterized protein *yhbO*	KpST66_0556	KPN_03561	Y/Y
	P024H5	33-kDa chaperonin	KpST66_0345	KPN_03772	Y/Y
	P007C4	putative α-helix	KpST66_0117	KPN_03990	N/Y
	P044A10	DNA helicase II	KpST66_4551	KPN_04312	Y/Y

**DNA**	P012H7	Cytoskeleton protein *rodZ*	KpST66_4055	KPN_02846	Y/Y
	P029G3	Putative DNA-processing protein	KpST66_0252	KPN_03865	Y/Y
	P043D4	Putative integrase	KpST66_0789	Unknown function	Y/Y
	P027A12	Putative Isrso16-transposase *orfb*	pKpST66–2_0103	Unknown function	Y/Y

**Hypotheticals**	P018D10	Putative lipoprotein	KpST66_3367	Unknown function*^b^*	Y/Y
	P044H7	Hypothetical CDS	KpST66_0778	Unknown function *^b^*	Y/Y
	P024E6	Putative uncharacterized protein	KpST66_0788	Unknown function*^b^*	Y/Y
	P034C10	Putative uncharacterized protein	KpST66_4517	Unknown function*^b^*	Y/Y
	P037F10	Hypothetical CDS	pKpST66–1_0077	Unknown function*^b^*	Y/Y
	P001G5	Subtilisin-related serine protease	pKpST66–1_0111	Unknown function*^b^*	Y/Y
	P054G10	Putative uncharacterized protein	KpST66_0786	Unknown function	Y/Y
	P043E4	Hypothetical protein	KpST66_3558	Unknown function	Y/Y
	P053H2	Putative uncharacterized protein	KpST66_2293	KPN_01157	Y/Y
	P013G3	Putative uncharacterized protein	KpST66_2888	KPN_01793	Y/Y
	P020B12	Uncharacterized protein conserved	KpST66_3673	KPN_02457	Y/Y
	P043G3	Cyclic diguanylate phosphodiesterase domain protein	KpST66_0830	KPN_03274	Y/Y
	P043G2	Putative uncharacterized protein	KpST66_0943	KPN_03171	Y/Y
	P025G5	Putative uncharacterized protein	KpST66_0942	KPN_03172	Y/Y
	P045H10	Hypothetical CDS	pKpST66–1_0020	KPN_pKPN3p05993	Y/Y
	P034F4	Hypothetical CDS	pKpST66–2_0092	KP1_p117	Y/Y
	P043D5	Putative uncharacterized protein	pKpST66–2_0058	LV102a (pLVPK)	Y/Y

**Non-coding regions**	P007G6	Between C4-type zinc finger protein, DksA/TraR family and GTP cyclohydrolase I	KpST66_3567 and KpST66_3568	Unknown function	Y/Y
	P009H6	Between LuxR-transcriptional regulator and a hypothetical protein	KpST66_1827 and KpST66_1828	Between KPN_00784 and KPN_00785	Y/Y
	P048H7	Between putative glutathione *S*-transferase and d-alanyl-d-alanine carboxypeptidase; penicillin-binding protein	KpST66_1922 and KpST66_1923	Between KPN_00870 and KPN_00871	Y/Y
	P043E6	Between *deoR* and *deoC*	KpST66_2811 and KpST66_2812	Between KPN_01701 and KPN_01702	Y/Y
	P038G8	Between aspartate-semialdehyde dehydrogenase and a putative transcriptional regulator	KpST66_2862 and KpST66_2863	Between KPN_01773 and KPN_01774	Y/Y
	P019E6	Between putative transposase IS4 and a putative ARAC-type regulatory protein	KpST66_2869 and KpST66_2870	Between KPN_01779 and KPN_01780	Y/Y
	P037E9	Between putative FAD/FMN-containing dehydrogenases and a hypothetical protein	KpST66_3144 and KpST66_3145	Between KPN_02042 and KPN_02043	Y/Y
	P022H9	Between putative enzyme and hypothetical protein	KpST66_3460 and KpST66_3461	Between KPN_02355 and KPN_02356	Y/Y
	P025H3	Between putative transcriptional regulator and putative arylsulfatase regulator	KpST66_4042	Between KPN_02830 and KPN_02831	Y/Y
	P050B12	Between *iap* and *cysH*	KpST66_0997 and KpST66_0998	Between KPN_03116 and KPN_03117	Y/Y
	P005D9	Between *uppP* and *folB*	KpST66_0645 and KpST66_0646	Between KPN_03461 and KPN_03462	Y/Y
	P015G5	Between two hypothetical proteins	KpST66_0630 and KpST66_0631	Between KPN_03477 and KPN_03478	Y/Y
	P006F8	Between *yhdP* and *rng*	KpST66_0465 and KpST66_0466	Between KPN_03654 and KPN_03655	Y/Y
	P028H3	Between *ibpA* and *yidQ*	KpST66_0014 and KpST66_0015	Between KPN_04091 and KPN_04092	Y/Y
	P041H3	Between *rimI* and *ygjF*	KpST66_5016 and KpST66_5017	Between KPN_04415 and KPN_04416	Y/Y
	P024D12	Between *yhaM* and *psiE*	KpST66_5012 and KpST66_5013	Between KPN_04419 and KPN_04420	Y/Y
	P051G11	Between *phnA* and *proP*	KpST66_4928 and KpST66_4929	Between KPN_04502 and KPN_04503	Y/Y
	P014F6	Between *YgfA* and *traM*	pKpST66–1_0029 and pKpST66–1_0030	Between KPN_pKPN3p06000 and KPN_pKPN3p06001	Y/Y
	P014E6	Between hypothetical protein and *iutA*	pKpST66–2_0063 and pKpST66–2_0064	Between LV106-LV107	Y/Y

*^a^* Growth in M9 agar plates was supplemented with either glucose or citrate as the carbon source. N, no growth; Y, growth.

*^b^* Present only in the Kp52145 genome.

Transposon insertion sites were determined by genomic sequencing using primer tpnRL17. The sequences obtained were compared with the Kp52145 genome and plasmid sequences (GenBank^TM^ accession numbers FO834904, FO834905, and FO834906) and to the reference sequence of *K. pneumoniae* strain MGH78578 (GenBank^TM^ accession number CP000647.1). Ninety-nine insertions were found in the chromosome, whereas nine and six were located in plasmid 1 and plasmid 2, respectively. The MicrobesOnline and STRING databases were interrogated to annotate the loci. Thirty of them were related to metabolism, twenty were included in the category outer membrane and envelope-related genes, nine in transport, eight in regulation of transcription, seven in the stress response category, and four in the category of DNA-related processes (Table 2). Interestingly, six of the insertions were located in regions of the Kp52145 genome not present in other *Klebsiella* genomes (27). Nineteen mutants had the transposon inserted in non-coding regions of the genome, and 17 were located within the coding region of hypothetical proteins.

In summary, our high-throughput genetic screen mining a *K. pneumoniae* transposon library led to the identification of 114 mutants that triggered the activation of NF-κB. In the following sections we describe the characterization of the mutants on the enterobactin siderophore, WaaL encoding the O-antigen ligase and pullulanase T2SS. We selected the enterobactin and ligase mutants because iron-scavenging systems and LPS are two well characterized *Klebsiella* virulence determinants, although their contributions to attenuating inflammation have not been evaluated previously. On the other hand, we chose to investigate the *Klebsiella* pullunase T2SS because its role in *Klebsiella* virulence had not been established.

##### Analysis of the Enterobactin Mutant

**FIGURE 3.**
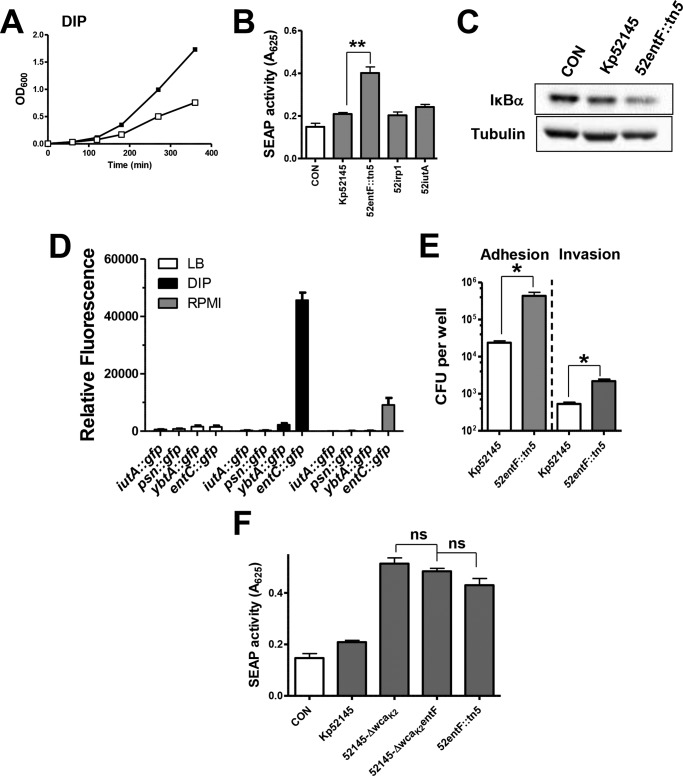
**Indirect role of *K. pneumoniae* enterobactin in attenuation of NF-κB activation.** *A*, growth of Kp52145 (*black symbols*) and enterobactin mutant (52entF::tn*5*, *white symbols*) in LB supplement with 2-2′-dipyridyl (*DIP*). *B*, SEAP levels released by A549-SEAP A2 cells left untreated (control (*CON*)) or infected with different *Klebsiella* strains. *Scale bars* represent mean ± S.E. (*n* = 3). *C*, immunoblot analysis of IκBα and tubulin levels in A549 cells left uninfected (control) or infected with Kp52145 or enterobactin mutant (52entF::tn5) for 3 h. Data are representative of three independent experiments. *D*, analysis of the expression of *iutA*, *psn*, *ybtA*, and *entC* by Kp52145 carrying the transcriptional fusions *iutA*::*gfp, psn*::*gfp*, *ybtA*:;*gfp*, and *entC*::*gfp*. Bacteria were grown in LB (*white bars*), RPMI 1640 (*gray bars*), or LB supplement with 2-2′-dipyridyl (*DIP*, *black bars*). Data are presented as mean ± S.D. (*n* = 3). *E*, adhesion (*left bars*) and internalization (*right bars*) of *Klebsiella* strains to A549 cells (*n* = 3). *F*, SEAP levels released by A549-SEAP A2 cells left untreated (control, *white bar*) or infected with Kp52145, capsule mutant (52145-Δ*wza*_K2_), enterobactin mutant (52entF::tn*5*), or capsule and enterobactin double mutant (52145-Δ*wza*_K2_entF). *Scale bars* represent mean ± S.E. (*n* = 3). *ns* (not significant), *p* > 0.05; *, *p* < 0.05; **, *p* < 0.01 (one-tailed *t* test).

One transposon insertion was identified in the enzyme EntF, a component of the biosynthetic machinery of the enterobactin siderophore (44). Enterobactin is one of the three siderophores expressed by Kp52145 (27). Enterobactin is expressed in the lungs of infected mice, and an enterobactin mutant is attenuated (9). As anticipated (9), the doubling times of the *entF* mutant slowed when grown in LB supplemented with the iron chelator 2-2′-dipyridyl (Fig. 3*A*). Similar rates of growth were observed when the wild type and the mutant were grown in LB or RPMI 1640 (data not shown).

We sought to determine whether the other two siderophores encoded by Kp52145, aerobactin and yersiniabactin, are implicated in attenuation of inflammation. However, the SEAP levels induced by the aerobactin mutant, strain 52iutA, and the yersiniabactin mutant, strain 52irp1, were not significantly different than those induced by the wild-type strain (Fig. 3*B*). In contrast, SEAP levels induced by the *entF* mutant were higher than those induced by Kp52145, which in turn, were not significantly different than those found in the supernatant of non-infected cells (Fig. 3*B*). In the canonical NF-κB activation pathway, nuclear translocation of NF-κB is preceded by phosphorylation and subsequent degradation of IκBα (45). Immunoblot analysis demonstrated that the *entF* mutant induced the degradation of IκBα in A549 cells (Fig. 3*C*).

These findings suggested that enterobactin, but not the other siderophores, was expressed under our infection conditions. To monitor the transcription of the siderophores quantitatively, transcriptional fusions were constructed in which a promoterless *gfp* gene was under the control of the *iutA*, *psn*, *ybtA*, and *entC* promoter regions (see “Experimental Procedures”). *iutA* has been used previously to study aerobactin expression and *psn* and *ybtA* to analyze yersiniabactin expression, whereas *entC* has been used to determine enterobactin expression (9). *gfp* fusions were introduced into Kp52145, and fluorescence was determined after growing the reporter strains in LB, LB supplemented with iron chelator 2-2′-dipyridyl, or RPMI 1640 (Fig. 3*D*). The *entC*::*gfp* fusion was the only one expressed under iron-limited conditions, LB plus 2-2′-dipyridyl and RPMI 1640 (Fig. 3*D*). It has been reported previously that bacteria up-regulate iron-scavenging systems when grown in tissue culture medium (46). Further confirming this finding, the addition of FeCl_3_ to RPMI 1640 decreased the expression of the *entC*::*gfp* fusion by 75% (9106 ± 850 RFU and 2190 ± 323 RFU, respectively; *p* < 0.05, one-tailed *t* test).

Collectively, our data are consistent with a model in which *K. pneumoniae* experiences a poor iron environment when infecting A549 cells, hence leading to the activation of the siderophore enterobactin. There are reports suggesting that siderophores may inhibit immune responses (47), hence making it possible that enterobactin may directly attenuate NF-κB activation. However, it has been also shown that *K. pneumoniae* CPS expression is down-regulated in iron-poor media (48). Therefore, the involvement of enterobactin in attenuating NF-κB activation would be indirect considering that CPS is known to prevent NF-κB activation (20, 21). Indirectly supporting the latter evidence, the *entF* mutant expressed 50% less cell-bound CPS than the wild-type strain (6.11 ± 2.99 mg/10^7^ cells and 11.49 ± 1.91 mg/10^7^ cells, respectively; *p* < 0.05, one-tailed *t* test), and the activity of the transcriptional *cps*::*lucFF* fusion was 80% lower in the *entF* mutant background than in the wild-type one (3.1 × 10^6^ ± 16,000 RLU and 1.7 × 10^7^ ± 34,000 RLU, respectively; *p* < 0.01, one-tailed *t* test). Further confirming the inverse correlation between the CPS levels and the adhesion and internalization to epithelial cells (49), the *entF* mutant adhered to and was internalized by A549 cells in higher numbers than the wild type (Fig. 3*E*). Altogether, this evidence suggests that *entF*-induced NF-κB activation could be explained by the reduced CPS levels expressed by the mutant. To validate this hypothesis, we asked whether the SEAP levels induced by the *entF* mutant were significantly different than those triggered by the *cps* mutant or the double *cps-entF* mutant. The data shown in Fig. 3*F* demonstrate that indeed there were no differences in the SEAP levels triggered by any of the three strains.

In aggregate, our findings revealed that enterobactin was activated under our tissue culture infection conditions and that the lack of the siderophore was linked to a reduced CPS expression. In turn, the low CPS levels underlined the NF-κB activation induced by the *entF* mutant.

##### Analysis of the LPS Polysaccharide Mutants

**FIGURE 4.**
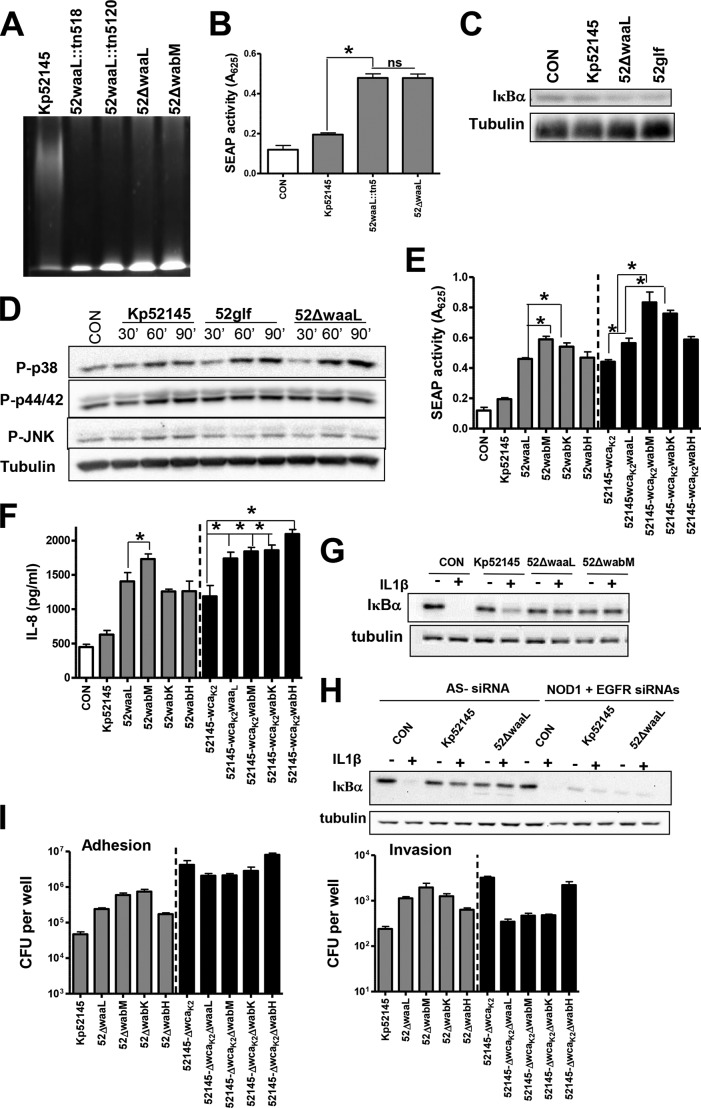
***K. pneumoniae* LPS O-polysaccharide is implicated in attenuation of NF-κB activation.** *A*, LPS analysis by SDS-PAGE. Data are representative of three independent experiments. *B*, SEAP levels released by A549 cells left untreated (control (*CON*), *white bar*) or infected with Kp52145 or *waaL* mutants (52waaL::tn5 and 52ΔwaaL). *Scale bars* represent mean ± S.E. (*n* = 3). *C*, immunoblot analysis of IκBα and tubulin levels in A549 cells left uninfected (control) or infected with the indicated strains for 3 h. Data are representative of three independent experiments. *D*, immunoblots showing phosphorylated MAPKs and tubulin levels in cell extracts of A549 cells left uninfected (control, time 0) or infected with *K. pneumoniae* strains for different times. The results are representative of three independent experiments. *E*, SEAP levels released by A549 cells left untreated (control, *white bar*) or infected with the indicated strains (*n* = 3). *F*, ELISA of IL-8 released by A549 cells left untreated (control, *white bar*) or infected for 12 h with the indicated strains (*n* = 3). *G* and *H*, immunoblot analysis of IκBα and tubulin levels in lysates of A549 cells infected with the indicated strains. The cells were left untreated, stimulated with IL-1β (1 ng/ml, 10 min), infected for 3 h, or infected for 3 h and then stimulated with IL-1β. In *H*, the siRNA targets are indicated in the *top row* of *each panel*. (AS−, AllStars control siRNA). Data are representative of three independent experiments. *I*, adhesion (*left panel*) and internalization (*right panel*) of *Klebsiella* strains to A549 cells (*n* = 3). *, *p* < 0.05 (one-tailed *t* test); *ns* (not significant), *p* > 0.05 (one-tailed *t* test). In *E* and *F*, CPS-expressing strains, *gray bars*; CPS negative strains, *black bars*.

Two transposon insertions were identified in the *waaL* gene. WaaL is the O-antigen ligase responsible for ligating the LPS O-polysaccharide (OPS) to the core (50). LPS analysis confirmed that neither of the mutants express OPS, and the LPS pattern was similar to that displayed by a nonpolar *waaL* mutant (50) (Fig. 4*A*). *waaL* is encoded within the operon responsible for the synthesis of the LPS core (50), hence making it possible that the transposon insertion would affect the expression of downstream genes. However, RT-qPCR experiments showed that the expression of the downstream gene *wabM* was not significantly different between the wild type and the transposon mutants (data not shown). WabM encodes the glycosyltransferase, adding the first glucose residue of the core where the OPS is attached (50). The SEAP levels induced by the ligase mutants were significantly higher than those induced by the wild type (Fig. 4*B*). No significant differences were found in the SEAP levels induced by any of the mutants (Fig. 4*B*).

To rule out the possibility that the non-ligated OPS exerted an unanticipated effect on *Klebsiella* physiology, we tested the *glf* glycosyltransferase mutant, strain 52glf. Glf is a UDP-galactopyranose mutase essential for OPS biosynthesis (51). Immunoblot analysis revealed that the ligase and OPS mutants triggered the degradation of IκBα in A549 cells in contrast to the wild-type strain (Fig. 4*C*). Many cellular stimuli also activate MAPK pathways. The activation of the three MAPKs (p38, JNK, and p44/42) occurred through phosphorylation of serine and threonine residues. All strains induced the phosphorylation of the three MAPKs; however the phosphorylation of p38 and p44/42 was more apparent in cells infected with the ligase and OPS mutants than in those infected with the wild type (Fig. 4*D*). No differences were observed between both mutants (Fig. 4, *C* and *D*). Altogether, these results support the view that Kp52145 OPS is required to attenuate inflammation.

*waaL* mutants express similar levels of CPS as the wild type (Ref. 50 and data not shown). To assess the relative contribution of CPS and OPS to attenuate NF-κB activation, we investigated whether the absence of OPS increases SEAP levels induced by the *cps* mutant. Results shown in Fig. 4*E* confirmed that this was the case.

To further analyze the contribution of the LPS polysaccharide section to the attenuation of NF-κB activation, we sought to determine whether the LPS core section plays any role in *Klebsiella* attenuation of NF-κB activation. *wabM*, *wabK*, and *wabH* mutants lack, in addition to the OPS, the first, second, and third sugars of the LPS core, respectively (50), but they express similar levels of CPS as the wild type (50). The three mutants triggered the secretion of SEAP by infected cells (Fig. 4*E*). SEAP levels were higher than those induced by the wild type; the levels induced by the *wabM* and *wabK* mutants were also significantly higher than those induced by the *waaL* mutant (Fig. 4*E*). No significant differences were found between the *wabM* and *wabK* mutants (Fig. 4*E*). In the genetic background of the *cps* mutant, the *wabM* and *wabK* mutants induced similar levels of SEAP, which were higher than those triggered by the *waaL* mutant (Fig. 4*E*). SEAP levels induced by the *wabH* mutant were not significantly different than those induced by the *waaL* mutant (Fig. 4*E*). Comparatively similar results were found when the secretion of IL-8 was evaluated (Fig. 4*F*). Collectively, these data show that the CPS, the OPS, and the first glucose of the core are bacterial factors required to attenuate the activation of NF-κB by epithelial cells upon *K. pneumoniae* infection.

Recently, we have shown that Kp52145 attenuates proinflammatory mediator-induced NF-κB activation (22). This process requires bacteria-cell contact, and removal of bacteria by washing followed by a 1-h gentamicin treatment renders the cells responsive to agonist-induced IL-8 secretion (22). To exert this anti-inflammatory effect, Kp52145 engages NOD1 and EGFR receptors (22, 24). To explore whether the OPS could account for the Kp52145 anti-inflammatory effect, we determined the effect of the *waaL* mutant on IL-1β-induced IκBα degradation using the assay that we described previously (22, 24). Similar to Kp52145, *waaL* attenuated IL-1β-induced IκBα degradation (Fig. 4*G*). As with the wild type, this effect was abrogated in cells in which NOD1 and EGFR were knocked down using siRNA (Fig. 4*H*).

Control experiments revealed that the ligase and core mutants adhered to A549 cells in higher numbers than the wild type (for each comparison between Kp52145 and the mutants, *p* < 0.05 (one-tailed Student's *t* test)) (Fig. 4*I*). The adhesion levels of the *wabM* and *wabK* mutants were higher than that of the *waaL* mutant (Fig. 4*I*). As reported previously (49), strains lacking CPS adhered in higher numbers than CPS-expressing strains (for each comparison between CPS-expressing and CPS-negative strains, *p* < 0.01 (one-tailed Student's *t* test)) (Fig. 4*I*). In the *cps* mutant background, no significant differences in adhesion between strains were found (Fig. 4*I*). *waaL* and core mutants were internalized in higher numbers than the wild type (for each comparison between Kp52145 and the LPS mutants, *p* < 0.01 (one-tailed Student's *t* test)) (Fig. 4*I*). In the *cps* mutant background, however, *waaL, wabM*, and *wabK* mutants were internalized in lower numbers than the *cps* mutant (Fig. 4*I*), and the levels were not significantly different than those of the wild type (for each comparison between Kp52145 and the LPS mutants, *p* > 0.05 (one-tailed Student's *t* test)). Interestingly, *wabH* mutant displayed internalization levels similar to those of the *cps* mutant (Fig. 4*I*).

**FIGURE 5.**
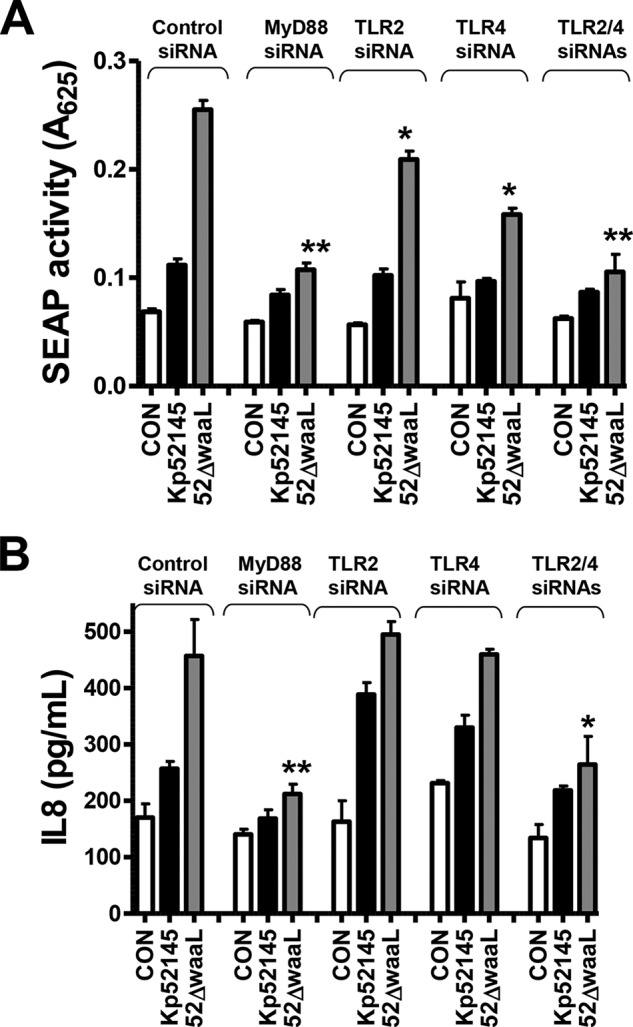
**Role of MyD88, TLR2, and TLR4 in *K. pneumoniae waaL* mutant-induced cell activation.** *A*, SEAP levels released by A549 cells transfected with the indicated siRNAs for different pattern recognition receptors, which were left untreated (control (*CON*), *white bars*) or infected with Kp52145 or *waaL* mutant (*n* = 3). *B*, ELISA of IL-8 released by A549 cells transfected with either control or the indicated siRNA for different pattern recognition receptors, which were left untreated (control, *white bars*) or infected (*n* = 3). *Scale bars* represent mean ± S.E. **, *p* < 0.01; *, *p* < 0.05 (results are significantly different from the results obtained by infecting control siRNA-transfected cells with *waaL* mutant; one-way ANOVA).

Finally, we sought to determine the innate receptor(s) involved in the recognition of the OPS mutant by using as cellular readouts of the NF-κB-dependent secretion of SEAP and the secretion of IL-8. Almost all TLRs activate cellular signaling pathways through TIR domain-mediated interactions with the adaptor molecule MyD88 (52). To explore the involvement of TLRs in *waaL* mutant-induced cell activation, the function of the MyD88 adaptor molecule was interrupted by siRNA. In MyD88 knocked-down cells, *waaL* mutant induced neither the activation of the NF-κB reported construct nor the secretion of IL-8 (Fig. 5, *A* and *B*, respectively). To further dissect the contribution of TLR-dependent signaling to *waaL* mutant-induced cell activation, TLR2 and TLR4 were knocked down by siRNA. The results shown in Fig. 5 indicate that both receptors contributed to *waaL*-induced cell activation. On the whole, these data suggest that *waaL* mutant induced NF-κB activation and IL-8 secretion is mediated by TLR4-TLR2-MyD88 pathway.

##### Analysis of the Pullulanase Mutants

**FIGURE 6.**
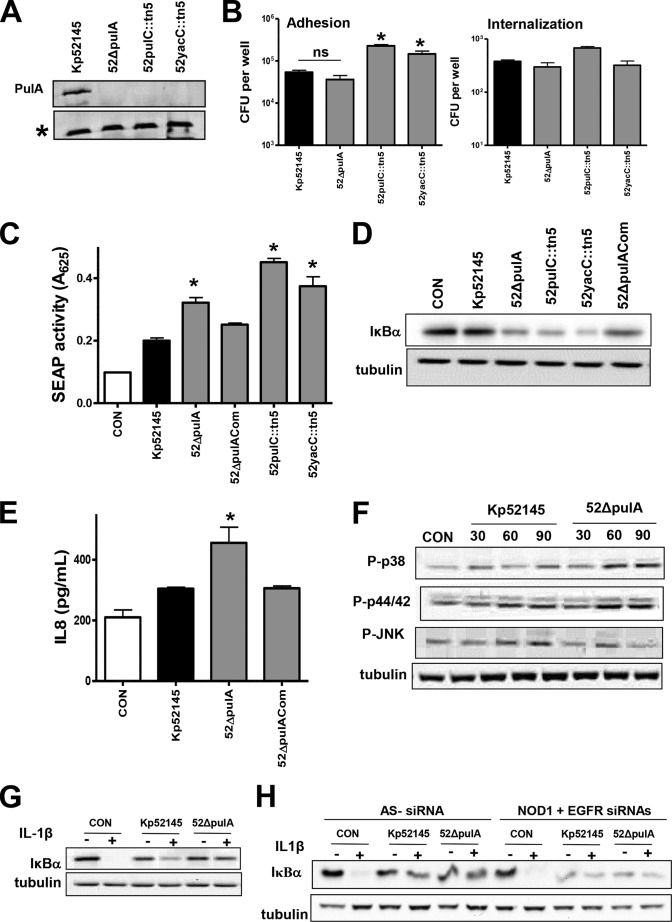
***K. pneumoniae* PulA T2SS is required for attenuation of NF-κB activation.** *A*, immunoblot analysis of PulA levels in the outer membranes of *Klebsiella* strains. An *asterisk* marks a protein also recognized by the anti-PulA antibody, which served as a loading control. Data are representative of three independent experiments. *B*, adhesion (*left panel*) and internalization (*right panel*) of *Klebsiella* strains to A549 cells (*n* = 3). *C*, SEAP levels released by A549 cells left untreated (control (*CON*), *white bar*) or infected with the indicated strains. *Scale bars* represent mean ± S.E. (*n* = 3). *D*, immunoblot analysis of IκBα and tubulin levels in A549 cells left uninfected (control) or infected for 3 h. Data are representative of three independent experiments. *E*, ELISA of IL-8 released by A549 cells left untreated (control, *white bar*) or infected for 12 h with the indicated strains (*n* = 3). *F*, immunoblots showing phosphorylated MAPKs and tubulin levels in cell extracts of A549 cells left uninfected (control, time 0) or infected with *K. pneumoniae* strains for different times. The results are representative of three independent experiments. *G* and *H*, immunoblot analysis of IκBα and tubulin levels in lysates of A549 cells infected with the indicated strains. The cells were left untreated, stimulated with IL-1β (1 ng/ml, 10 min), infected for 3 h, or infected for 3 h and then stimulated with IL-1β. In *H*, siRNA targets are indicated in the *top row* of *each panel* (AS−, AllStars control siRNA). Data are representative of three independent experiments. *, *p* < 0.05 (results are significantly different from the results for Kp52145; one-tailed *t* test). *ns* (not significant), *p* > 0.05 (for the indicated comparisons; one-tailed *t* test).

Two transposon insertions were found in the *pulC* and loci. *pulC* is the first locus of the *Klebsiella* T2SS that secrets the enzyme pullulanase encoded by *pulA* (53). *yacC* encodes for a lipoprotein containing a domain related to the *pulS/outS* family but for which the exact function has not yet been described. To help understand the contribution of *Klebsiella* T2SS to NF-κB attenuation, we constructed a *pulA* mutant, because PulA is the only known protein secreted by *K. pneumoniae* T2SS (54). Immunoblot analysis confirmed the absence of the enzyme pullulanse in the outer membranes of the *pulC* and *yacC* mutants and, as expected, also in the outer membrane of the *pulA* mutant (Fig. 6*A*). The amount of cell-bound CPS expressed by the mutants was quantified, and no differences were found between the CPS expressed by the wild type (11.49 ± 1.91 mg/10^7^ cells) and the CPS expressed by any of the mutants (52ΔpulA, 15.21 ± 3.65 mg/10^7^ cells; 52pulC::tn*5*, 9.44 ± 2.91 mg/10^7^ cells; and 52yacC::tn*5*, 13.26 ± 4.97 mg/10^7^ cells; for each comparison between wild-type CPS levels and mutant levels, *p* > 0.05, one-tailed Student's *t* test). Control experiments revealed that the adhesion levels of the *pulC* and *yacC* mutants were higher than those of the *pulA* and Kp52145 strains, which in turn, were not significantly different (Fig. 6*B*). In contrast, no significant differences were found in the internalization to cells between any of the strains (Fig. 6*B*).

SEAP levels induced by the T2SS mutants were higher than those induced by the wild type (Fig. 6*C*), and IκBα levels were lower in those cells infected with the T2SS mutants (Fig. 6*D*). Complementation of the *pulA* mutant restored the SEAP levels to those triggered by the wild type, and we found an increase in the levels of IκBα in the lysates of infected cells (Fig. 6, *C* and *D*). Cells infected with the *pulA* mutants also secreted higher levels of IL-8 than cells infected with the wild type (Fig. 6*E*). Complementation of the mutant restored IL-8 secretion to wild-type levels. We also evaluated the activation of MAPKs in *pulA* mutant-infected cells. Western blot analysis shown in Fig. 6*E* revealed that infection with the *pulA* mutant triggered the phosphorylation of the three MAPKs. Phosphorylation levels of p38 and p44/42 were higher at 60 and 90 min post-infection in cells infected with the *pulA* mutant than in those infected with Kp52145 (Fig. 6*E*). Kp52145 and *pulA* mutant triggered similar levels of phosphorylated JNK (Fig. 6*E*). In aggregate, these findings indicate that *K. pneumoniae* that PulA contributes to limiting the activation of the NF-κB canonical pathway and MAPKs p38 and p44/42.

We next sought to clarify whether T2SS-secreted PulA is required for *K. pneumoniae* anti-inflammatory phenotype (22, 24). Similar to Kp52145, *pulA* mutant attenuated IL-1β-induced IκBα degradation (Fig. 6*G*). Furthermore, this effect was dependent on NOD1 and EGFR, because the *pulA* mutant did not block cytokine-triggered IκBα degradation in NOD1-EGFR knockdown cells (Fig. 6*H*).

**FIGURE 7.**
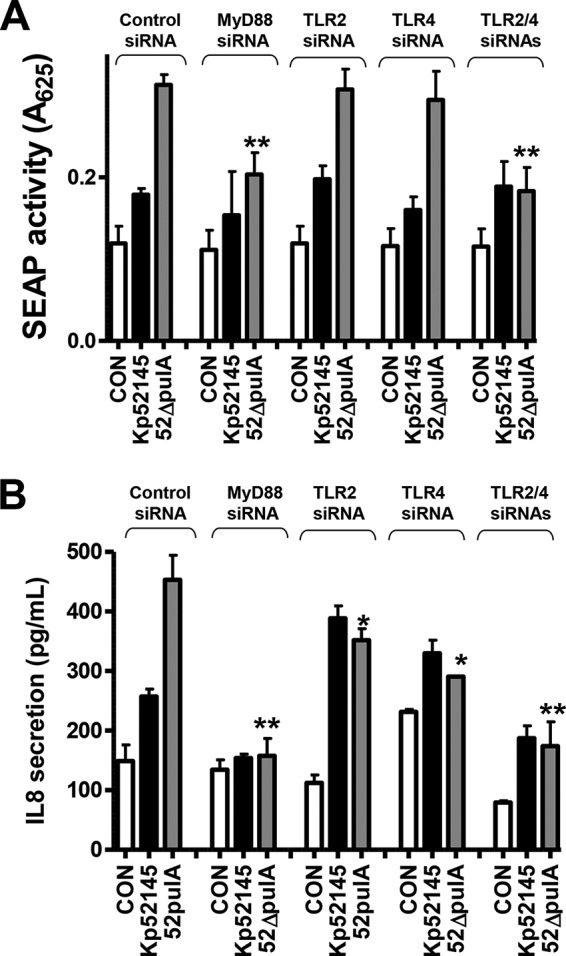
**Role of MyD88, TLR2, and TLR4 in *K. pneumoniae pulA* mutant-induced cell activation.** *A*, SEAP levels released by A549 cells transfected with the indicated siRNAs for different pattern recognition receptors, which were left untreated (control (*CON*), *white bars*) or infected with Kp52145 or *pulA* mutant (*n* = 3). *B*, ELISA of IL-8 released by A549 cells transfected with either control or the indicated siRNA for different pattern recognition receptors, which were left untreated (control, *white bars*) or infected (*n* = 3). *Scale bars* represent mean ± S.E. **, *p* < 0.01; *, *p* < 0.05 (results are significantly different from the results by obtained infecting control siRNA-transfected cells with *pulA* mutant; one-way ANOVA).

siRNA-based experiments were carried out to investigate whether a TLR-MyD88-dependent pathway is implicated in the recognition of the *pulA* mutant. SEAP levels induced by the *pulA* mutant were reduced to those induced by the wild type only in MyD88 knocked-down cells and in TLR2-TLR4 knocked-down cells (Fig. 7*A*). Likewise, *pulA* mutant-triggered IL-8 secretion was also dependent on TLR4-TLR2-MyD88 (Fig. 7*B*). Altogether, our results demonstrated that *K. pneumoniae* PulA is required to attenuate the activation of NF-κB by limiting the activation of a TLR4-TLR2-MyD88 pathway.

##### Relative Contribution of CPS, O-polysaccharide, and pulA to K. pneumoniae Attenuation of Inflammation

**FIGURE 8.**
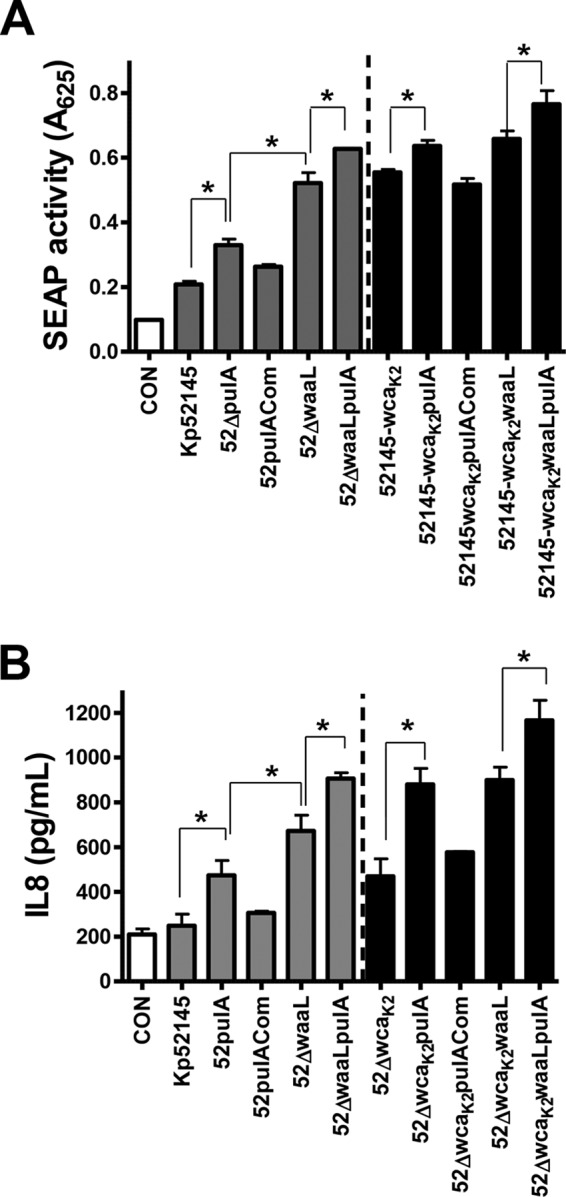
**Relative contribution of CPS, LPS O-polysaccharide, and PulA T2SS to *K. pneumoniae*-triggered attenuation of NF-κB activation.** *A*, SEAP levels released by A549 cells left untreated (control (*CON*), *white bar*) or infected with the indicated strains (*n* = 3). *B*, ELISA of IL-8 released by A549 cells left untreated (control, *white bar*) or infected for 12 h with the indicated strains (*n* = 3). *Scale bars* represent mean ± S.E. CPS-expressing strains, *gray bars*; CPS negative strains, *black bars*. *, *p* < 0.05 (for the indicated comparisons, one-tailed *t* test).

The fact that CPS, LPS OPS, and PulA are all involved in attenuating inflammatory responses led us to study the relative contribution of each of these factors. Therefore, we asked whether the absence of PulA further increases the SEAP levels and the secretion of IL-8 induced by the *cps* and *waaL* mutants. Data displayed in Fig. 7*A* show that SEAP levels induced by the double mutant *waaL-pulA* were higher than those induced by either the *waaL* or *pulA* single mutant. In turn, *waaL*-infected cells secreted higher SEAP levels than cells infected with the *pulA* mutant (Fig. 8*A*). In the *cps* mutant background, the strain inducing the highest SEAP levels was the double mutant *waaL-pulA*. No significant differences were observed between the *waaL* and *pulA* mutants. Similar picture was obtained when the IL-8 secretion was used as read out for cellular activation (Fig. 8*B*). Collectively, these data show that the CPS, the LPS OPS and PulA are *Klebsiella* factors require to limit NF-κB activation and the secretion of IL-8 by infected epithelial cells.

##### Virulence of K. pneumoniae Mutants

**FIGURE 9.**
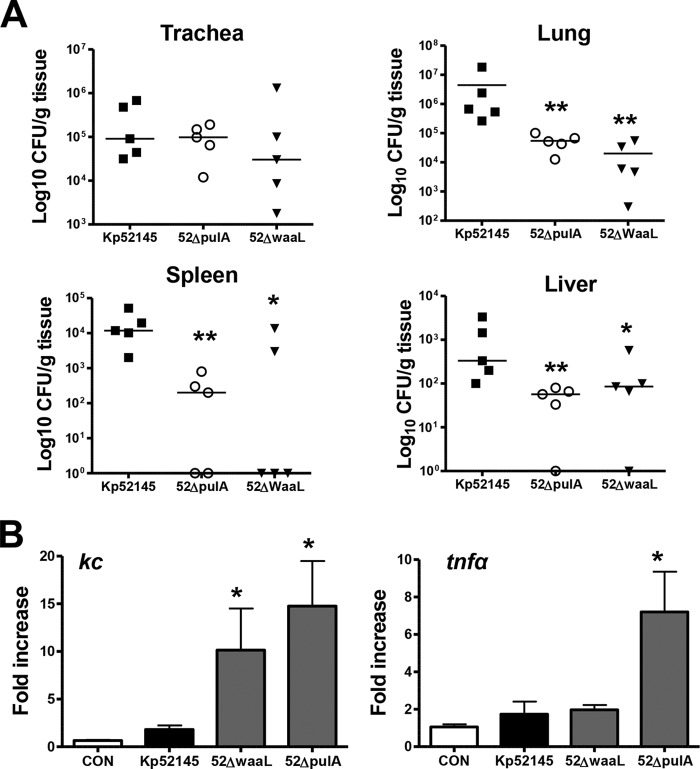
**Virulence of *K. pneumoniae waaL* and *pulA* mutants.** *A*, bacterial counts in mouse organs at 24 h post-infection. Mice were infected intranasally with the indicated strains (Kp52145, ■), *pulA* mutant (52ΔpulA, ○) or *waaL* mutant (52ΔwaaL, ▾). Results were reported as log cfu/g of tissue. **, *p* < 0.01; *, *p* < 0.05 (results are significantly different from the results obtained infecting with Kp52145; one-way ANOVA). *B*, *kc* and *tnf*α mRNA expressions in whole lungs were assessed by RT-qPCR. Mice were non-infected (control (*CON*), *white bars*, *n* = 5) or infected with Kp52145 (*black bars*, *n* = 5) or *waaL* and *pulA* mutants (*gray bars*; *n* = 5 for each mutant). *Scale bars* represent mean ± S.E. *, *p* < 0.05 (results are significantly different from the results obtained by infecting with Kp52145; one-way ANOVA).

To determine the ability of *pulA* and *waaL* mutants to cause pneumonia, CD-1 mice were infected intranasally, and 24 h post-infection the bacterial loads in trachea, lung, spleen, and liver homogenates were determined. No differences were found in the bacterial loads in the trachea (Fig. 9*A*). In contrast, the bacterial loads of the *pulA* and *waaL* mutants were significantly lower in lung, spleen, and liver than those of the wild type (Fig. 9*A*).

We also assessed by RT-qPCR the expression levels of *kc* and *tnf*α cytokines in the lungs of infected mice. The levels of *kc* were higher in lungs of infected mice than in lungs of non-infected animals (*p* < 0.05 for all comparisons *versus* non-infected mice; one-tailed *t* test) (Fig. 9*B*). However, *kc* levels were significantly higher in the lungs of mice infected with *pulA* and *waaL* mutants than in those infected with the wild type (Fig. 9*B*). *tnf*α levels were higher in the lungs of mice infected with *pulA* mutant than in those infected with the wild type or the *waaL* mutant.

## Discussion

We recently showed that *K. pneumoniae* dampens the activation of inflammatory responses in host cells by targeting the activation of the NF-κB canonical pathway (22, 24). Our results revealed that *K. pneumoniae* CPS is necessary but not sufficient to attenuate inflammation (22, 24). To identify additional *Klebsiella* factors required to dampen inflammation, we standardized and applied a high-throughput gain-of-function screen, mining a *Klebsiella* transposon mutant library. Using this approach, we identified 114 mutants that triggered the activation of NF-κB. Follow-up validation uncovered that, in addition to the CPS, *K. pneumoniae* LPS OPS and the pullulanase T2SS are required for evasion of innate immune responses. Table 3 summarizes the findings obtained in this study. This study represents the first functional genomics-driven identification of previously unknown bacterial factors required to down-regulate the NF-κB signaling pathway.

**TABLE 3:** **Summary of the phenotypes displayed by the *K. pneumoniae* mutants characterized in this study** NT, not tested; —, no capsule; ∼, similar to the wild-type strain; ↑, higher/stronger than the wild type, where the number of arrows gives an indication of relative levels compared with the wild type; ↓, less than the wild type.

*Klebsiella* factor	Phenotype of the mutants						
	Adhesion/internalization	CPS levels	NF-κB activation*^a^*	MAPK activation*^b^*			TLR signaling*^c^*
				p38	p44/42	JNK	
**Siderophores**							
*entF*	↑	↓	↑↑		NT		NT
*cps-entF*	NT	—	↑↑		NT		NT
*iutA*	NT	NT	∼		NT		NT
*Irp1*	NT	NT	∼		NT		NT

**LPS**							
*waaL*	∼	∼	↑	↑	↑	∼	Yes
*glf*	∼	∼	↑	↑	↑	∼	NT
*wabM*	∼	∼	↑↑		NT		NT
*cps-waaL*	∼	—	↑↑		NT		NT
*cps-wabM*	↑	—	↑↑↑↑		NT		NT

**T2SS**							
*pulA*	∼	∼	↑	↑	↑	∼	Yes
*cps-pulA*	NT	—	↑↑		NT		NT
*cps-waaL-pulA*	NT	—	↑↑↑↑		NT		NT

*^a^* SEAP levels as read-out.

*^b^* Phosphorylation of the MAPKs as read-out.

*^c^* IL-8 secretion and SEAP levels are dependent on the TLR4-TLR2-MyD88 signaling pathway.

The high number of *Klebsiella* loci involved in the attenuation of NF-κB activation was somewhat unanticipated. To the best of our knowledge the majority of the loci identified in our screening have never been implicated in NF-κB control, and even the function of most of them is largely speculative, as it is based on *in silico* predictions. Nevertheless, it should be noted that the transposon mutant library examined is not saturated, hence making it possible that additional loci are needed for fully effective immune evasion. For example, in our screening we did not retrieve any *cps* mutant, despite the fact that CPS plays a crucial role in *Klebsiella* anti-immune strategies (8, 20, 24). However, this is not unprecedented in this type of studies; for example, *cps* mutants were not isolated in other *Klebsiella* screenings searching for virulence factors (4, 55, 56).

The two gene ontology categories including half of the loci identified in the screening are: metabolism and transport, 32% of the mutants, and envelope-related genes, 17% of the mutants. None of the metabolic and transport mutants displayed any growth defects under the screening conditions or in LB, hence making it unlikely that any gross growth defect underlies the activation of NF-κB in infected cells. As this will be the subject of future studies, at present we speculated that their contribution to *Klebsiella* immune evasion is indirect and related to their impact on other *Klebsiella* factors. Supporting this hypothesis, the follow-up analysis of the enterobactin mutant revealed that the lack of the siderophore is linked to a reduced CPS expression, which in turn explains the NF-κB activation induced by the mutant. The regulatory connection between iron levels and CPS expression has been already established (48). Because our wild-type strain encodes for two additional iron-scavenging systems, yersiniabactin and aerobactin, we sought to determine whether any *Klebsiella* siderophore mutant would induce NF-κB activation. However, and in good agreement with Lawlor *et al*. (9), enterobactin was the only siderophore expressed under our infection conditions. Therefore, it was not unexpected to find that the mutants in the other two scavenging systems did not activate NF-κB. Nonetheless, the evidence indicates that yersiniabactin is the *Klebsiella* siderophore that plays a dominant role *in vivo* (9), hence making possible that yersiniabactin would be the siderophore implicated in *Klebsiella* immune evasion during pneumonia.

The other major set of mutants were related to envelope structures, thereby adding further evidence to the critical role played by bacterial surface structures on host-pathogen interactions. Although in this work we have characterized only the role of the OPS and T2SS systems, it is worth discussing the possible contribution of the peptidoglycan and adhesion mutants to *Klebsiella* immune evasion. The seminal discoveries showing that NOD receptors recognize peptidoglycan motifs from Gram-negative bacteria provide a mechanistic explanation for the observation that peptidoglycan mutants induce NF-κB activation (57, 58). This is because these mutants secrete peptidoglycan fragments into the medium, which then activate NOD1 (59). Future efforts in our laboratory will confirm whether this is the case with our set of mutants. In addition, we will be eager to explore whether *K. pneumoniae* remodels its peptidoglycan to attenuate detection by NOD receptors.

In our previous studies we did demonstrate that *Klebsiella*-cell contact is essential to attenuate NF-κB activation (22). In this scenario, it can be predicted that any mutant deficient in adhesion will be affected on its ability to block NF-κB activation. Of note, in our recent siRNA-based screening we uncovered the fact that *Klebsiella* may manipulate the β1-integrin-ILK signaling cascade to dampen inflammatory responses (24). β1-integrins are used by many pathogens as cellular receptors for attachment (60). It is then tempting to speculate that the adhesion-related mutants found in the present study might not be able to target the β1-integrin-ILK signaling cascade.

The LPS OPS is perhaps the most widely found factor in any screen designed to identify virulence factors of Gram-negative bacteria. However, to the best of our knowledge, ours is the first screening to demonstrate its involvement in attenuating NF-κB activation. The fact that the double mutant lacking CPS and OPS induced higher responses than each of the single mutants indicates that the *Klebsiella* polysaccharides do not play redundant roles in host-*Klebsiella* interactions. Having established the role of the OPS, we next investigated whether LPS core residues may also participate in immune evasion. To investigate the contribution of core residues in an OPS-bearing strain, we used defined mutants that lack the OPS in addition to core residues, which in turn might suggest that the core residues will never be exposed in a wild-type strain. However, it should be noted that epidemiological data indicate that nearly 10% of *Klebsiella* clinical isolates do not express the LPS OPS (61), and therefore, core residues will not be masked by the OPS. Our results revealed that the first glucose residue of the LPS core is necessary to attenuate NF-κB activation, because the SEAP levels induced by the *wabM* mutant, lacking also OPS, were higher than those triggered by the OPS mutant. Elimination of additional core residues did not further increase the SEAP levels.

The invasion experiments revealed unexpected features of the contribution of CPS and LPS to *Klebsiella* interaction with epithelial cells. Available evidence supports the notion that *Klebsiella* CPS is the critical factor preventing *Klebsiella* internalization by epithelial cells (49). Our data further corroborated this notion but also highlighted the contribution of OPS and core residues. However, in the absence of CPS, the d-galactan OPS mediates *Klebsiella* internalization by epithelial cells because the internalization of those mutants lacking the OPS, mutants *waaL*, *wabM*, and *wabK*, was reduced to the levels of the wild-type strain. Surprisingly, the internalization levels of the *wabH* mutant were similar to those of the CPS mutant. Structural studies showed that a core galactose residue gets exposed in this mutant (50), which in turn could account for the mutant internalization.

The contribution of the LPS core to virulence is poorly characterized in most Gram-negative pathogens, and it has been rigorously established only for *Yersinia enterocolitica* and Kp52145 (50, 62). In a previous study we uncovered the contribution of the *Klebsiella* core in preventing macrophage phagocytosis (6), which, together with the evidence presented in this work, led us to put forward the notion that core residues are important for *Klebsiella* immune evasion. Interestingly, this might be a general feature of LPS core sections, as it has been reported recently that *Brucella abortus* core acts as a shield against immune recognition (63). Future studies testing additional pathogens should help to further validate this hypothesis.

In this work, we discovered that *Klebsiella* pullulanase T2SS is important for immune evasion *in vitro* and *in vivo*. The *Klebsiella* pullulanase secretion system is the archetype of T2SS, and most of the previous research efforts have been devoted to characterizing the molecular structure and function of the secretion system (54). However, its contribution to *Klebsiella* virulence, if any, was unknown. Perusal of the literature indicates that PulA is the only protein secreted by *K. pneumoniae* T2SS, although presently we cannot rigorously rule out that other proteins yet to be found are also secreted by the system. Nonetheless, the fact that SEAP levels induced by the *pulA*, *pulC*, and *yacC* mutants were not significantly different may support the idea that indeed the only protein secreted by the T2SS is PulA. Taking into account the evidence available for similar enzymes from other pathogens (64, 65), we speculate that PulA interacts with glycan(s) located in the epithelial surface, hence leading to limited activation of the TLR4-TLR2-MyD88 pathway upon *Klebsiella* infection. Future efforts of the laboratory will be devoted to identify these host glycan(s).

In this work, we have demonstrated for the first time, using a pneumonia mouse model, that PulA is important for bacterial survival in the lung. Research over the last 20 years has demonstrated a correlation between the activation of inflammatory responses and *Klebsiella* clearance from the airways (11–14). Hence the higher inflammatory response induced by the *pulA* mutant may contribute to mutant clearance. It should be noted that cytokines and chemokines released by epithelial cells do increase the bactericidal activity of professional phagocytes. Studies are ongoing to determine whether PulA plays any role in the interaction of *K. pneumoniae* with alveolar macrophages and neutrophils.

Recently, we demonstrated that *K. pneumoniae* exerts an anti-inflammatory effect by engaging NOD1 and EGF receptors (22, 24). Bacterial removal by gentamicin treatment renders cells again responsive to pro-inflammatory mediators (22). Considering that CPS is necessary but not sufficient to attenuate inflammation (22, 24), in this work we asked whether OPS and PulA could mediate this anti-inflammatory effect. However, our findings shown in Figs. 4 and 6 do not support this. These results do not contradict the other findings reported in this work showing that OPS and T2SS mutants activate inflammatory responses. It should be noted that in most of the experiments in this study gentamicin was used to remove bacteria after a 2-h infection; for example, IL-8 in the supernatants was measured at 12 h post-infection. In turn, the fact that *waaL* and *pulA* mutant-induced responses were dependent on TLR2-TLR4-MyD88 activation suggests that OPS and PulA perturb TLR-dependent recognition of *K. pneumoniae*. These results further corroborate *in vitro* and *in vivo* data showing that TLR2 and TLR4 play a dominant role in detecting *K. pneumoniae* infections (20, 66, 67).

On the whole, the evidence is consistent with the notion that an essential aspect of *K. pneumoniae* infection biology is to thwart the TLR-dependent activation of host defense responses. Our studies have revealed the concerted action of CPS, OmpA, LPS polysaccharide, and T2SS to prevent TLR-dependent responses (this work and others (8, 20, 21)). Further highlighting the importance of immune evasion for *Klebsiella* virulence, all of these mutants are attenuated in the pneumonia model (this work and others (3, 8)). Therefore, we put forward the notion that a new therapeutic approach to the treatment of *Klebsiella* infections will be to prevent this virulence strategy, either by using molecules directed to block some of these factors or by increasing TLR-governed defense responses. Supporting the feasibility of the latter approach, the administration of the TLR ligand CpG augments anti-*Klebsiella* immunity in pneumonia (17).

## Footnotes

3 The abbreviations used are:

CPS: capsule polysaccharide
EGFR: EGF receptor
Omp: outer membrane protein
TLR: Toll-like receptor
OPS: O-polysaccharide
T2SS: type 2 secretion system
SEAP: secreted form of the human embryonic alkaline phosphatase
RLU: relative light unit
RT-qPCR: real time quantitative PCR
ANOVA: analysis of variance
Cm: cloramphenicol
Tp: trimethoprim
RFU: relative fluorescence units.
